# Supplementary material for: What promotes or prevents greater use of appropriate compression in people with venous leg ulcers? A qualitative interview study with nurses in the north of England using the Theoretical Domains Framework
Source: BMJ Open. 2022 Aug 1;12(8):e061834. doi: 10.1136/bmjopen-2022-061834 (PMC9345063; doi:10.1136/bmjopen-2022-061834)
Supplement: Supplementary data [file bmjopen-2022-061834supp001.pdf]

Barriers and facilitators to compression therapy for people with venous leg ulcers – staff interview schedule

As part of this project we're really interested in understanding the barriers and facilitators to the delivery of compression therapy for people with venous leg ulcers, including factors that may operate at an individual, team and organisation level. We are also interested in your perspectives on how the behaviours of patients themselves might impact positively or negatively on compression use.

Question	Prompts	Rationale/Theoretical Domains Framework (TDF) domains
**Can you tell me a little bit about your current role and your main responsibilities?**	• *How long have you been in this role?*<br>• *What did you do before?*<br>• *How is your team/clinic set up and organised?*	Easy first question, scene setting, supplements demographic information supplied, may reveal something important in relation to role which can be probed later (e.g. 'go to' leg ulcer person).
**From your perspective, is there a 'typical' leg ulcer patient? Could you describe them to me?**	• *Age/sex?*<br>• *Co-morbidities?*<br>• *Lifestyle factors?*	Scene setting.
**Can you tell me what 'gold standard' care and treatment for a patient with a venous leg ulcer looks like?**	• *Vascular assessment.*<br>• *Cleansing and debridement.*<br>• *Dressings and topical treatments.*<br>• *Compression therapy.*<br>• *Systemic therapy.*<br>• *Provision of care.*	Establishing something about interviewees' knowledge/experience – the background against which they practice.<br><br>TDF: knowledge; professional role and identity.
**What guides your choices about venous leg ulcer care?**	• *Knowledge and skills – training, peers, experience, preferences?*<br>• *Local policies?*	TDF: knowledge; skills; professional role and identity; beliefs about capabilities; optimism; beliefs about consequences;

Perry C, *et al. BMJ Open* 2022; 12:e061834. doi: 10.1136/bmjopen-2022-061834

**Question**	**Prompts**	**Rationale/Theoretical Domains Framework (TDF) domains**
	• *Research evidence - Scottish Intercollegiate Guidelines Network (SIGN) guidelines, Cochrane reviews, reading journals, online searches, specialist peers?*<br>• *Patient and carers' influence – lifestyle, preference, adherence.*	reinforcement; intentions; memory, attention and decision processes; environmental context and resources; social influences; emotion; behavioural regulation.
**What is your experience of offering and delivering compression therapy to patients with venous leg ulcers?**	• *How important do you think compression is?*<br>• *What guides your decision to offer compression? What are your thought processes?*<br>• *How do you explain it to patients and what information do you routinely give them (any patient information/leaflets given)?*<br>• *Do you think patients understand the importance of it?*<br>• *Is there a particular type of compression you like?*<br>• *Do you start on full compression and reduce if patient cannot tolerate/start low and work up to full? Why?*<br>• *How easy or difficult do you find it to deliver compression therapy?*<br>• *What do you think are the necessary skills for effective compression delivery?*<br>• *Have you received relevant training to deliver compression therapy?*<br>• *Are there times when you would choose not to offer or deliver compression (even if it was clinically indicated)?*	TDF: knowledge; skills; professional role and identity; beliefs about capabilities; optimism; beliefs about consequences; reinforcement; intentions; goals; memory, attention and decision processes; environmental context and resources; social influences; emotion; behavioural regulation.
**I'd like to ask you specifically about what you see as the barriers to offering and delivering compression therapy, and what the facilitators are.**<br><br>**Are barriers and facilitators to offering compression therapy different to those**	***Individual level***<br>• *Do you personally find it easy/difficult to offer and deliver compression therapy?*<br>• *How important do you think the ABPI measurement is?*<br>• *What do you do if you are looking after a patient who has not had an ABPI measurement taken?*<br>• *How confident are you in using a Doppler?*	TDF: knowledge; skills; beliefs about capabilities; emotion.

Perry C, *et al. BMJ Open* 2022; 12:e061834. doi: 10.1136/bmjopen-2022-061834

Question	Prompts	Rationale/Theoretical Domains Framework (TDF) domains
**of delivering compression therapy (may want to address separately if so)?**<br><br>**It may be easier to look at barriers and facilitators in turn. Barriers? Facilitators?**	• *How confident are you in offering and delivering compression therapy?*<br>• *Is there anything that would make you more confident?*<br><br>***Team level***<br>• *Would any other team members influence whether or not you offer or deliver compression therapy (who and how)?*<br>***Organisational level***<br>• *Are there aspects of the community/clinic environment which influence whether or not you are able to offer and deliver compression?*<br>• *Are there any time constraints that influence whether or not you are able to offer and deliver compression?*<br>• *Are there any competing tasks that influence whether or not you are able to offer and deliver compression?*<br>• *Are there any specific issues (e.g. staffing) that influence whether or not you are able to offer and deliver compression?*<br>*If yes to any of the above – how big an issue is this, how does this issue affect your decision making, do you use simpler techniques/interventions so that less experienced staff can then continue using them?*<br>• *What systems are in place to assist with compression use (how easy or difficult is it to deliver compression)?*<br>• *What availability is there of Dopplers; staff training; patient information leaflets?*	TDF: professional role and identity; reinforcement; social influences.<br>TDF: professional role and identity; reinforcement; intentions; environmental context and resources; social influences; behavioural regulation.
**How might the views of your patients affect how you offer and deliver compression?**	• *Does compression therapy influence your relationships with patients? (does it affect communication?)*<br>• *How do you respond when a patient refuses to wear compression/is non-adherent?*<br>• *Do your colleagues generally agree with you about this approach to patient non-adherence?*	TDF: beliefs about consequences; goals; environmental context and resources; social influences; emotion.

Question	Prompts	Rationale/Theoretical Domains Framework (TDF) domains
	• *Do you find that you change from full compression to reduced compression?*<br>• *What are your views about reduced compression?*<br>• *Do you find patients adhere to reduced compression?*<br>• *Does not being able to deliver compression cause you to be worried or concerned?*	
**Have you any views on why patients do or do not adhere to their compression therapy?**	• *Patient discomfort?*<br>• *Patient lifestyle?*<br>• *Patient beliefs about illness and treatment?*<br>• *Influence of partner/family/friends?*<br>• *Do your colleagues generally agree with you about patient non-adherence?*	TDF: beliefs about consequences; goals; environmental context and resources; social influences; emotion.
**What do you perceive to be the consequences of offering and delivering compression therapy?**	• *Does it influence the outcomes?*<br>• *Does it have an impact on your clinical workload and patient routines?*<br>• *What happens if you are not able to deliver compression as you would like to? (to patients, to colleagues, to yourself? Short and long-term consequences?)*	TDF: beliefs about consequences; optimism; intentions; goals; social influences; emotion.
**What would help you to overcome any difficulties/challenges that you perceive in offering and delivering compression therapy?**	• Skills training in using a Doppler machine?<br>• Other skills training?<br>• The number of Dopplers available?<br>• Other equipment?<br>• *What would you personally have to do to increase the number of patients wearing compression? (in the ideal world).*<br>• *If you wanted to implement changes in your own practice to increase the number of patients wearing compression, what would be some of the ways to do this?*	TDF: knowledge; skills; beliefs about capabilities; optimism; reinforcement; memory, attention and decision processes; environmental context and resources; behavioural regulation.

Perry C, *et al. BMJ Open* 2022; 12:e061834. doi: 10.1136/bmjopen-2022-061834

Question	Prompts	Rationale/Theoretical Domains Framework (TDF) domains
**Is there anything else about your experience of offering and delivering compression therapy that I haven't asked you about that you think it is important to say?**		Tapping into knowledge/experience of individual.
**Is there anybody else that you can think of (individual/groups) who you think it might be useful for me to talk to as part of this study?**		Snowballing/tapping into knowledge/experience of individual.

Perry C, et al. BMJ Open 2022; 12:e061834. doi: 10.1136/bmjopen-2022-061834
