# Supplementary material for: What promotes or prevents greater use of appropriate compression in people with venous leg ulcers? A qualitative interview study with nurses in the north of England using the Theoretical Domains Framework
Source: BMJ Open. 2022 Aug 1;12(8):e061834. doi: 10.1136/bmjopen-2022-061834 (PMC9345063; doi:10.1136/bmjopen-2022-061834)
Supplement: Author's manuscript [file bmjopen-2022-061834.draft_revisions.pdf]

BMJ Open is committed to open peer review. As part of this commitment we make the peer review history of every article we publish publicly available.

When an article is published we post the peer reviewers' comments and the authors' responses online. We also post the versions of the paper that were used during peer review. These are the versions that the peer review comments apply to.

The versions of the paper that follow are the versions that were submitted during the peer review process. They are not the versions of record or the final published versions. They should not be cited or distributed as the published version of this manuscript.

BMJ Open is an open access journal and the full, final, typeset and author-corrected version of record of the manuscript is available on our site with no access controls, subscription charges or pay-per-view fees (http://bmjopen.bmj.com).

If you have any questions on BMJ Open's open peer review process please email info.bmjopen@bmj.com

BMJ Open

What promotes or prevents greater use of appropriate compression in people with venous leg ulcers? A qualitative interview study with nurses using the Theoretical Domains Framework

Journal:	*BMJ Open*
Manuscript ID	bmjopen-2022-061834
Article Type:	Original research
Date Submitted by the Author:	08-Feb-2022
Complete List of Authors:	Perry, Catherine ; The University of Manchester, Division of Population Health, Health Services Research and Primary Care, School of Health Sciences<br>Atkinson, Ross; The University of Manchester, Division of Nursing, Midwifery and Social Work, School of Health Sciences, Faculty of Biology, Medicine and Health<br>Griffiths, Jane; The University of Manchester, Division of Nursing, Midwifery and Social Work, School of Health Sciences<br>Wilson, Paul; University of Manchester, Division of Population Health, Health Services Research and Primary Care, School of Health Sciences<br>Cullum, Nicky; The University of Manchester, School of Health Sciences; Manchester University NHS Foundation Trust, MAHSC<br>Lavallée, Jacqueline; The University of Manchester, Division of Medical Education, School of Medical Sciences<br>Mullings, Julie; Manchester University NHS Foundation Trust<br>Dumville, Jo C.; The University of Manchester, Division of Nursing, Midwifery and Social Work, School of Health Sciences
Keywords:	Vascular medicine < INTERNAL MEDICINE, PRIMARY CARE, QUALITATIVE RESEARCH, WOUND MANAGEMENT

SCHOLARONE™
Manuscripts

I, the Submitting Author has the right to grant and does grant on behalf of all authors of the Work (as defined in the below author licence), an exclusive licence and/or a non-exclusive licence for contributions from authors who are: i) UK Crown employees; ii) where BMJ has agreed a CC-BY licence shall apply, and/or iii) in accordance with the terms applicable for US Federal Government officers or employees acting as part of their official duties; on a worldwide, perpetual, irrevocable, royalty-free basis to BMJ Publishing Group Ltd ("BMJ") its licensees and where the relevant Journal is co-owned by BMJ to the co-owners of the Journal, to publish the Work in this journal and any other BMJ products and to exploit all rights, as set out in our licence.

The Submitting Author accepts and understands that any supply made under these terms is made by BMJ to the Submitting Author unless you are acting as an employee on behalf of your employer or a postgraduate student of an affiliated institution which is paying any applicable article publishing charge ("APC") for Open Access articles. Where the Submitting Author wishes to make the Work available on an Open Access basis (and intends to pay the relevant APC), the terms of reuse of such Open Access shall be governed by a Creative Commons licence – details of these licences and which Creative Commons licence will apply to this Work are set out in our licence referred to above.

Other than as permitted in any relevant BMJ Author's Self Archiving Policies, I confirm this Work has not been accepted for publication elsewhere, is not being considered for publication elsewhere and does not duplicate material already published. I confirm all authors consent to publication of this Work and authorise the granting of this licence.

For peer review only

What promotes or prevents greater use of appropriate compression in people with venous leg ulcers? A qualitative interview study with nurses using the Theoretical Domains Framework

Authors

Catherine Perry, Division of Population Health, Health Services Research and Primary Care, School of Health Sciences, University of Manchester, UK.
(Corresponding author: Catherine.perry@manchester.ac.uk)

Ross A Atkinson, Division of Nursing, Midwifery and Social Work, School of Health Sciences, University of Manchester, UK.

Jane Griffiths, Division of Nursing, Midwifery and Social Work, School of Health Sciences, University of Manchester, UK.

Paul M Wilson, Division of Population Health, Health Services Research and Primary Care, School of Health Sciences, University of Manchester, UK.

Nicky Cullum, Division of Nursing, Midwifery and Social Work, School of Health Sciences, University of Manchester, and MAHSC, Manchester University NHS Foundation Trust, Manchester, UK.

Jacqueline F Lavallée, Division of Medical Education, School of Medical Sciences, University of Manchester, UK.

Julie Mullings, Manchester University NHS Foundation Trust, Manchester, UK.

Jo C Dumville, Division of Nursing, Midwifery and Social Work, School of Health Sciences, University of Manchester, UK.

Disclaimer

The views expressed in this article are those of the authors and not necessarily those of the NHS, National Institute for Health Research, or the Department of Health and Social care.

Ethics approval

Ethical approval was received in 2019 from the London Bridge NHS Research Ethics Committee (Ref 19/LO/0826).

For peer review only - http://bmjopen.bmj.com/site/about/guidelines.xhtml

Patient consent for publication

Not required.

Word count

6083

For peer review only

For peer review only - http://bmjopen.bmj.com/site/about/guidelines.xhtml

What promotes or prevents greater use of appropriate compression in people with venous leg ulcers? A qualitative interview study with nurses using the Theoretical Domains Framework

ABSTRACT

Objectives

To investigate factors that promote and prevent the use of compression therapy in people with venous leg ulcers.

Design

Qualitative interview study with nurses using the Theoretical Domains Framework.

Setting

Three NHS Trusts in the North of England.

Participants

Purposive sample of 15 nurses delivering wound care from participating Trusts.

Findings

Nurses described factors which they perceived made provision of compression therapy challenging. Organisational barriers included heavy/increasing caseloads and pressure of work; issues related to the knowledge and skills of healthcare practitioners and the provision of training (particularly time to attend); and issues related to prescribing (variations in bandaging systems used and whether nurses could prescribe compression). Absence of specialist leg ulcer services to which they could refer patients was perceived as a barrier to providing optimal leg ulcer care by some community nurses. Patient engagement barriers focussed on the importance of getting patients ‘on board’ with compression therapy, and supporting them in using it. Clear explanations were seen as key.

Conclusions

Rising patient caseloads and related workload pressures present significant challenges to any attempt to enhance existing leg ulcer services. There may be opportunities to develop facilitated approaches to enable community nursing teams to make changes to practice and enhance quality of patient care. The majority of venous leg ulcers can arguably be managed in general community services without referral to a specialist community service if issues relating to workloads, skills and training in the community are addressed. Barriers in relation to promoting appropriate compression use could also usefully be targeted, for example through the development of clear patient information leaflets. Whilst patient engagement barriers may be easier/quicker to address than some organisational barriers, unless organisational barriers are also attended to it seems unlikely that all people who would benefit from compression therapy will receive it.

For peer review only - http://bmjopen.bmj.com/site/about/guidelines.xhtml

Strengths and limitations of this study

- Using qualitative interviews with nurses delivering wound care enabled an in-depth exploration of what promotes or prevents the use of compression therapy in people with venous leg ulcers, from the perspective of staff.
- Initial inductive analysis of interview data ensured that all data were accounted for, and the use of the Theoretical Domains Framework provided a theoretical structure for understanding factors that promoted or prevented compression us.
- Participating nurses were from three Trusts (provider organisations) in the North of England, staff in other geographical regions may have other views.
- All interviews were conducted over the telephone due to the COVID-19 pandemic, so those less happy with using the telephone may not have participated.

For peer review only

INTRODUCTION

Background to this study

Venous leg ulcers (VLUs) are common, open wounds on the lower leg which take more than two weeks to heal.[1] Problems with the veins in the leg (such as damage to the valves, or blockages), or impaired action of the calf muscles, prevent optimal blood flow to the heart. This causes venous stasis, increasing the pressure in the veins and triggering a complex change in tissue physiology that can result in open wounds which heal slowly, if at all. Leg ulcers are more prevalent in older people. They can be painful, impair mobility and sleep, restrict work and leisure activities and reduce psychological well-being, impacting greatly on health-related quality of life.[2-4] Complete wound healing has been identified as the main outcome desired by people with VLUs.[5]

In the UK, VLU care is commonly delivered in the community, in patients' homes or clinics, by nurses or other health professionals. Whilst a wide range of largely nurse-delivered community-focused interventions are available to treat VLUs (bandages, hosiery, dressings and topical agents among others), only compression therapy is supported by a convincing level of evidence[6] and recommended by clinical guidelines.[7 8] Compression therapy, in the form of bandages or stockings, is cost-effective and has become the first-line, evidence-based treatment for VLUs.[6 9] In addition, compression hosiery is current standard practice for prevention.[10]

Compression works when used as intended, but delivery and adherence can be problematic. For example, previous research has highlighted unwarranted variation in clinical practices across wound care services in the north of England.[11] This included significant underuse of

For peer review only - http://bmjopen.bmj.com/site/about/guidelines.xhtml

compression therapy where clinically appropriate. Understanding how and why sub-optimal delivery of evidence-based practices happens is an essential prerequisite to developing strategies to enhance the quality of care delivered. Whilst there is recognition that patient factors may play a role, including discomfort, patients' understanding of the causes of ulceration, and their beliefs about compression in terms of healing and symptom control,[12, 13] less is known about the influences on professional behaviours. The aim of this study was to explore, from the perspective of nurses delivering wound care, the factors that promote or prevent the use of compression therapy for people with VLUs.

Theoretical underpinning

Understanding existing professional practices with a view to promoting positive changes in individual and collective behaviour requires an understanding of the influences on behaviours in the context in which they occur.[14] The Theoretical Domains Framework (TDF) is a comprehensive, theory-informed approach for identifying determinants of professional behaviour (cognitive, affective, social and environmental).[11 12] Organised across 14 conceptual domains (see Table 1), the TDF has been applied across several healthcare settings to explore professional practices and behaviours.[14] We used the TDF to investigate the factors that promote and prevent the use of compression therapy as a treatment for VLUs; thus identifying potentially modifiable determinants of implementation and providing a theoretical foundation to link these to targets for behaviour change.

For peer review only - http://bmjopen.bmj.com/site/about/guidelines.xhtml

Table 1 The Theoretical Domains Framework: domains and definitions[15]

Domain	Definition
Knowledge	An awareness of the existence of something
Skills	An ability or proficiency acquired through practice
Social/professional role and identity	A coherent set of behaviours and displayed personal qualities of an individual in a social or work setting
Beliefs about capabilities	Acceptance of the truth, reality, or validity about an ability, talent or facility that a person can put to constructive use
Optimism	The confidence that things will happen for the best or that desired goals will be attained
Beliefs about consequences	Acceptance of the truth, reality or validity about the outcomes of a behaviour in a given situation
Reinforcement	Increasing the probability of a response by arranging a dependent relationship or contingency between the response and a given stimulus
Intentions	A conscious decision to perform a behaviour or a resolve to act in a certain way
Goals	Mental representations of outcomes and states that an individual wants to achieve
Memory, attention and decision processes	The ability to retain information, focus selectively on aspects of the environment and choose between two or more alternatives
Environmental context and resources	Any circumstance of a person's situation or environment that discourages or encourages the development of skills and abilities, independence, social competence and adaptive behaviour
Social influences	Those interpersonal processes that can cause individuals to change their thoughts, feelings or behaviours
Emotion	A complex reaction pattern, involving experiential, behavioural, and psychological elements, by which the individual attempts to deal with a personally significant matter or event
Behavioural regulation	Anything aimed at managing or changing objectively observed or measured actions

For peer review only

For peer review only - http://bmjopen.bmj.com/site/about/guidelines.xhtml

METHODS

Sample

We purposively sampled participants from three NHS Trusts in the North of England. All were qualified nurses providing leg ulcer care within the community, including community nurses and nurses who worked in hospital outpatient clinics.

Participant recruitment and data generation

Recruitment and data generation took place between February 2020 and January 2021. Potential participants were contacted by their manager, who provided them with a brief description of the study and a link to an online form to register their interest in participating. Those completing the online form were contacted via email, including a Participant Information Sheet, by a researcher. If they were happy to participate a date and time were arranged for interview. Sampling was continued until it was judged that data saturation had been reached.[16]

We had planned to conduct face-to-face interviews, however, because of COVID-19 restrictions, all interviews were conducted over the telephone. Verbal consent was taken and digitally audio-recorded.

We undertook semi-structured interviews as they offer a good way to generate data on individuals' experiences.[17] We developed a semi-structured interview schedule (Supplementary File 1) with reference to the research aim, the literature, and the TDF. Interview questions defined the area to be explored[18] but allowed the interviewer or interviewee to diverge in order to follow up particular areas in more detail.[19] With the permission of participants, interviews were digitally audio-recorded and transcribed.

Data analysis

We uploaded interview transcripts onto NVivo software to aid data management.[20] Initially, an inductive thematic analysis was undertaken to create a framework for the data.[21] This was followed by a deductive analysis in which the data from the framework were mapped on to the TDF domains

The initial inductive analysis was carried out by the lead qualitative researcher who had conducted all of the interviews (CP) and to ensure dependability[22] the developing analytical framework was shared and discussed with the research team. Similarly, the deductive analysis mapping specific barriers and enablers within each TDF domain was carried out by one author (CP) in regular discussion with the research team, with additional sense-making

For peer review only - http://bmjopen.bmj.com/site/about/guidelines.xhtml

and insight provided by the Study Steering Group. To ensure credibility[22] a near final version of the analysis was presented to a group of nurses experienced in wound care from across England, who agreed that the findings (and our interpretation of them) reflected their own experiences and/or made sense to them.

Patient and public involvement

A patient representative sits on the Study Steering Group and has been an active member of the team, contributing to the work from the writing of the study protocol through to discussion of emerging findings and their interpretation. The study was also presented to the University of Manchester Wounds Research Patient and Public Involvement (PPI) Forum. In particular members of the forum were consulted about interview schedule questions covering issues pertinent to them, for example questions regarding the importance of the staff-patient relationship.

FINDINGS

Participant characteristics

We interviewed 15 nurses who delivered wound care, with interviews between 25 and 69 minutes in length. Interviewees were all female and comprised nine community nurses or managers, three vascular nurse specialists and three leg ulcer nurses, with a range of experience represented (Table 2).

Relevant domains and key themes

We coded data to seven TDF domains: environmental context and resources, goals, knowledge, skills, beliefs about consequences, behavioural regulation and intentions. The domains of knowledge and skills are presented together as they were linked in the data by the issue of training. Domains and their associated themes are displayed in Table 3. The findings are presented in relation to the TDF domains, with the domains to which most data were coded presented first. Quotations from participants are used to illustrate points made.

Table 3 Domains and associated themes

Domain	Theme
Environmental context and resources	Lack of commissioned leg ulcer services<br>Delays in initial leg and wound assessment<br>Lack of continuity of care<br>‘Joined up’ services and multidisciplinary teams<br>Prescribing issues<br>Application of compression as physically hard work

For peer review only - http://bmjopen.bmj.com/site/about/guidelines.xhtml

Goals	Getting patients 'on board'<br>Explaining timeframes around healing
Knowledge and skills	Gaps in knowledge and skills<br>Training
Beliefs about consequences	Encouraging compression use<br>Response to non-adherence
Behavioural regulation	Variations in bandaging systems used
Intentions	Local policies and leg ulcer pathways<br>Compression as treatment of choice

Environmental context and resources

Lack of commissioned leg ulcer services

In some areas the lack of specifically commissioned leg ulcer services was mentioned as a barrier to the provision of appropriate compression therapy; in these areas leg ulcer care was provided as part of general community nursing services: *'It's just wrapped up in district nursing.'* (S014). Community nurses discussed the challenge of offering optimal care, either because of perceived lack of skills in compression therapy or because of other responsibilities, resulting in apparently eligible people not receiving compression: *'You're not able to follow up….and like offer the treatment as they should anyway because of all the other things you have to worry about.'* (S002). Non-housebound patients were not eligible for home visits from community nursing services; in some areas they could attend community nurse-led clinics but in others they received leg ulcer care from practice nurses at their GP surgery, and these nurses were perceived as less likely to be trained in compression therapy: *'Sometimes the practice nurses are not competent to do the compression bandaging.'* (S004). These issues could affect the type of compression a patient was offered.

In areas with commissioned leg ulcer services and leg ulcer clinics, the clinics were highly thought of and seen as facilitating good quality care, incorporating appropriately trained staff and continuity:

> *'The leg ulcer clinics in [name of areas] are fabulous. Fabulous for patients, great for continuity, but also, great for anybody who has joined the district nursing team to make sure we've got that consistency across the* [area].*'* (S014).

For peer review only - http://bmjopen.bmj.com/site/about/guidelines.xhtml

Questions were, however, raised about the accessibility of some clinics, and it was suggested that patients may have difficulty getting to clinic locations on multiple/frequent occasions.

Delays in initial leg and wound assessment

The importance of fast, timely, initial assessment of someone with a suspected VLU was a universal theme: *'Getting the treatment in place quickly, because otherwise it can become….like the chronicity increases and the chance of healing decreases.'* (S004). However, delays in initial assessment were often described. This could be because community nurses did not recognise the need for specialised assessment of a wound: *'Nobody's thought about doing a Doppler. And that might be because they're not experienced, so hadn't thought about it.'* (S006); or that they lacked the skills to carry it out: *'There's only a select few of us within the team who can complete the Doppler assessment…..there's just not enough of us.'* (S005); (the Doppler assessment measures ankle-brachial pressure index (ABPI) and is an important component of initial assessment prior to compression use). Delays in access to Doppler were also ascribed to the general pressure of work in the community:

> *'So, pressure of work most definitely is a barrier……you could go in, see the patient…..you know put the dressing on. You are in and out of that house in 15 minutes, aren't you? You can also send your unregistered, or registered but non-nurse members of the team and so, even though our leg ulcer pathway, if you like, says, the gold standard, after two weeks you should be doing a Doppler, pressure of work, if you've got loads and loads of visits and not many staff and you've got low grades of staff, well then, that can slip can't it?'* (S014).

Even in areas with dedicated leg ulcer clinics delays in getting an initial assessment were described:

> *'There needs to be more of them* [leg ulcer clinics]*, because patients are still waiting too long for assessment. So we see lots of patients in vascular* [clinic] *that have waited three, four months. So they've been seeing the district nurses, waiting three or four months to go and see the leg ulcer clinic, and in that time their leg's getting worse and worse and worse, and in the end, the GP's referred them to Vascular. So they've ended up in secondary care, because the waiting lists in primary care are too long.'* (S003).

Two interviewees highlighted the value of specific assessment clinics which had been established to enable timely assessment and one spoke about a new role that she had undertaken providing community vascular support, which she said could lead to more timely assessments. More broadly, interviewees spoke about promoting awareness of leg care. One talked about *'a Legs Matter week….what they tend to do is promote healthy legs and*

For peer review only

promote compression and promote good skincare' (S006) and another suggested *'it's not just patients, just nurses in general need [awareness raising]'* (S001).

Lack of continuity of care

A lack of continuity of nursing care, with different staff attending patients, was perceived as a barrier to effective compression therapy. It was more a problem for community teams than in leg ulcer clinics, and could result in staff not having an overview of a patient's care, knowledge of their disease or treatment history, or reasoning behind clinical decisions. A nurse explained:

> *'The teams are so large, what will happen is, somebody will start the reduced compression. And then obviously, a different person goes in next time, and they will think they're in reduced compression, because they've got a problem with their arterial line. And no one will ever increase that compression, because they assume, because they're in reduced compression, that they've got some, you know…'* (S003).

This situation could be exacerbated by the lack of availability of electronic patient records in the community. A vascular clinic nurse commented:

> *'So that's another thing that adds to it. When everyone's on different [patient record systems]. "We can't access the [electronic] hospital records". I don't think the district nurses have got access. We have but they don't.'* (S010).

Lack of continuity in staffing was perceived as leading to a situation where *'somebody else thinks somebody else's, you know, it's somebody else's responsibility'* (S006). One interviewee described a change in working practices, 'zonal working' where a smaller group of nurses covered a defined geographical area, as increasing the likelihood of staff continuity. In addition, patients having contact details for the nursing team, for example through a telephone help-line, was thought to enhance continuity of care and communication between staff and patients.

'Joined up' services and multidisciplinary teams

A lack of communication between different services was identified as a barrier to optimal care. A patient with a VLU could be seen by hospital vascular services, leg ulcer clinics and district nursing services, as well as have contact with primary and secondary care services for issues other than their VLU. It was reported that these services did not necessarily communicate well with each other, resulting in unnecessary duplication (e.g., where a patient gets referred to *both* a leg ulcer clinic and a vascular clinic) or unnecessary referral (e.g., where a patient had undergone investigations whilst a hospital inpatient but the results

For peer review only

of these investigations were not communicated to the other services. Patients not receiving their compression therapy whilst hospital inpatients was frequently mentioned:

> *'Another barrier to our work is that we can be working really hard with a patient, they become unwell, they're admitted to hospital for something completely different. And the first thing they do is take the compression bandages off their legs and just stick a plaster on. Because nobody within hospital can do that treatment either.'* (S001).

Conversely, good liaison between vascular or leg ulcer clinics and community nursing teams was described as a facilitator of good care. Often a patient would be seen in a vascular or leg ulcer clinic, a treatment plan drawn up and they would be returned to community teams to continue the treatment. Both clinic-based nurses and community-based nurses reported good communication and liaison between the two: *'Any of the patients now that come to see me leave this clinic with a concise treatment plan…..I've got good established links with community'* (S003, clinic-based nurse); *'We have the numbers of all the vascular specialist nurses, so if we feel like we just need to ask a little bit of advice… they'll get back to us straight away'* (S013, community-based nurse).

Multidisciplinary teams, including nurses, physiotherapists, podiatrists and social workers working well together were described as facilitating high quality VLU care and compression therapy. The importance of working with the care system was also highlighted in terms of care workers being able to support patients with their compression therapy. One nurse also suggested working with care workers on training:

> *'The care providers, because they're often quite low-paid, so they have minimal training and that's why we're, our vision is joint training. Because, if they could come on our leg ulcer morning or updates, so that they understand the importance of the compression.'* (S014).

Prescribing issues

Compression therapy is a prescribed treatment. Nurses who had been trained as independent prescribers were able to prescribe compression, others had to ask a patient's GP to prescribe the treatment. This could cause delays and could result in patients not having prescribed treatment in time for their next nurse appointment:

> *'[Patient takes] your form to the GP. It has to sit at the GP's for 48 hours. They then do a prescription. The patient has to pick the prescription up, take it to the chemist. You might wait another 48 hours to pick your prescription up.'* (S001).

For peer review only - http://bmjopen.bmj.com/site/about/guidelines.xhtml

Most clinic nurses and some community nurses had stocks of compression which they could use if necessary (patients were often started off in their compression from these stocks), but this was not always the case.

Application of compression as physically hard work

Some interviewees reported that application of compression therapy, in some circumstances, can be physically hard work, particularly where the application takes place in patients' homes without any aids including treatment couches at the correct height. One interviewee commented: *'for district nurses it will often be on the floor on their hands and knees….so it is physically a little bit tricky'* (S004) and another: *'we've had a couple of staff that had got either bad backs or bad knees. So that takes them out of being able to do it for a couple of weeks'* (S012). Suggested facilitators were working in pairs, and spreading out compression visits over several days in order to avoid having too many on one day, but these solutions often depended on adequate staffing.

Goals

Getting patients 'on board'

Interviewees described the importance of getting patients 'on board' with their treatment, that is in agreement with and involved with it, from the beginning: *'I think if they're not on board from the beginning, you're fighting a losing battle.'* (S005). This could be difficult: *'Getting them on board to comply with the compression, and the correct compression. I think basically, that's the biggest one that we have a problem with.'* (S007). Explanations were seen as key to engaging patients: nurses were clear that if patients understood that their VLU was a symptom of an underlying long-term condition, and understood the mechanism of compression therapy, they were more likely to adhere to treatment.

In relation to explaining aetiology and compression therapy, interviewees talked about the availability and utility of written information to use with patients. A minority did not use written information at all and some thought that patients did not read information leaflets anyway. However, other interviewees thought that written information could be useful:

> *'So it just gives them something to read at their own leisure, and then next time you go, they've got a bit more of an understanding of what you're talking about as well. So I find the leaflets do work quite well.'* (S008).

Some interviewees had access to 'Trust leaflets' and product company leaflets were also mentioned, although worries were expressed about bias, which may be indicative of variation in the quality of this information. In order to be useful interviewees thought that

For peer review only - http://bmjopen.bmj.com/site/about/guidelines.xhtml

leaflets needed to have very simple, clear explanations, some said that pictures were helpful, and one person suggested alternative information formats such as short films.

In terms of getting patients 'on board', interviewees also spoke of giving patients options or choices, which might make them more likely to adhere to their compression therapy. One nurse explained:

> *'Like this is how I would feel you'd do best, however, these are your other options. And I'd rather give options at this point and formulate a plan, because you tend to get a little bit more concordance than saying well, this is what you are going to do.'* (S001).

There were a number of situations in which it could be possible to offer options, for example less obtrusive compression or being flexible with clinic appointments. Generally nurses agreed that it was important to build a relationship with their patient in which they could discuss treatment issues and work together towards the best outcome for the patient: *'A lot of it is getting them on board, building that relationship up with your patients…….and it's a journey together really.'* (S007); *'It's teamwork, it's not a one-way system.'* (S002).

Explaining timeframes around healing

The difficulty in estimating how long a VLU may take to heal and explaining this to a patient could be a barrier to setting goals and the successful use of compression therapy. All interviewees said that patients always asked about how long healing would take: *'I think that's one of the first things they'll ask you.'* (S001). Nurses suggested that giving a specific time frame should be avoided: *'The worst thing you could do is give the time. Because, then they're going to sit and wait and say right it's been this long, and it's not healed.'* (S008). A number of things that interviewees thought helped in explaining timeframes included being positive; talking about healing in small steps; describing what patients can do to speed up healing and issues that may slow healing; and being honest about ulcers that are likely to take a long time to heal, if at all.

One particular difficulty with trying to explain timeframes around healing was that in many cases nurses suggested healing of a VLU does not take a straightforward trajectory over time, and a wound may appear to get worse. Interviewees described patients becoming *'really disheartened'* (S005). There was consensus that there was usually a discernible cause for an ulcer deteriorating, thus the important thing was to identify the cause and address it: *'You can try and look at a reason why it has happened, like infection. And then treat that cause…….and tell them* [patient] *that you've addressed that so there is some hope for them that will work.'* (S004).

For peer review only - http://bmjopen.bmj.com/site/about/guidelines.xhtml

Knowledge and skills

Gaps in knowledge and skills

There is some suggestion that not all community nurses have the necessary knowledge to recognise the need for a comprehensive leg and wound assessment, or the necessary skills to carry out this initial assessment (including ABPI). Interviewees also recognised a lack of skills among some community nurses in relation to the application of compression bandages: *'The next district nurse that's gone in is not competent in bandaging, so they've not been able to receive the treatment.'* (S003); and the monitoring of compression therapy:

> '[Patient] *who I sent back to the district nurses to look after…..nobody had bothered to put it* [compression bandages] *back on because she didn't really want it on. So nobody had gone through all the things again, why you need to have it, why it's going to help you….nobody had bothered putting it back on because she'd said "Oh I don't really want it".' (S010).*

Training

Interviewees identified issues with the provision of training to address the gaps in knowledge and skills as being a barrier to the provision of compression therapy. Although training provision was generally considered to be adequate for those working in more specialised roles, for community nurses this was not always the case. A minority of interviewees thought that the availability of training was not good, and a majority mentioned access being more of an issue – it could be challenging to take time out to attend training because of workloads: *'The first thing that gets cancelled is training, unfortunately, 'cos obviously the needs of the patient come before training.'* (S005). It was also noted that staff turnover in the community was high, so there was always a need for training. Some positive examples of training were given; a community nurse talked about training offered by her Tissue Viability Team which she described as *'really good….we do it yearly, so we do have leg ulcer training yearly and compression training'* (005). Various facilitators of training were suggested, for example it was thought that provision of training 'on site' may get around issues of time to attend. Another interesting issue was the kind of 'informal' training that could be gained by nurses shadowing others in leg ulcer clinics, where these were available: *'You could go and spend an afternoon there and it's just that…..The experts are there, great.'* (S014).

Beliefs about consequences

Encouraging compression use

Interviewees thought that frequent reiteration of explanations about compression being the best therapy was important in encouraging patients to continue using it. They talked about patients being able to 'self-manage' their care and compression therapy as a goal to be

For peer review only - http://bmjopen.bmj.com/site/about/guidelines.xhtml

aimed for, which they perceived could contribute to the wider use of compression. However this *'can be difficult sometimes, but we're trying to encourage that self-caring as well and encourage them to take control over their care'* (S002). Barriers to self-care were thought to involve not only patients but the thinking and attitudes of nurses themselves:

> *'Self-management's quite a new…when I say new, I mean sort of, in the last few years. But it was always, sort of, the nurse came and did your dressing, and the nurse looked after it. And, I think, even for staff who've been around a long time it's hard getting that sort of, out of your head. So, it's about, sort of, changing your whole attitude and going in from the start and with the idea [that the patient will self-manage].'* (S006).

Response to non-adherence

Interviewees spoke about how their response to a patient who was not adhering to compression therapy was important in terms of whether that person might carry on, or would try compression again in the future: *'We have to ask why and is there anything else we can do to change that or make it better. Try and find the root of the cause of why they're saying no and then take it from there.'* (S002). It was also universally agreed that there will always be patients who will not tolerate or adhere to compression therapy, and that this was another point at which there was a need to be sensitive in responding in order to support the patient and keep the door open for them to try compression again in the future:

> *'I think if they are very sure they don't want to, and we do see…we do see a few like that, you just, I mean, you just kind of say that you understand their choice and, of course, it is their choice what they have on and we understand the reasons why. But that they have to also understand that, you know, healing will be delayed, or indeed will be very difficult to achieve without compression, in a nice way.'* (S004).

Behavioural regulation

Variation in available bandaging systems

Bandaging systems available for use varied geographically, as recommended by local Tissue Viability or Leg Ulcer Teams. When asked whether she was constrained in what compression therapy she could use for a patient based on where a patient lived and the bandage system used there one clinic-based nurse replied: *'I tend to because you just don't want to make life any more difficult'* (S003). Community nurses sometimes changed the compression type decided upon by specialist services because they are not able to provide it in their geographical area: *'We change them to* [name of compression brand] *because that's what we can…..that's what our ordering system allows us to order'* (S012).

Intentions

We did not map any barriers to this domain. Instead, most interviewees talked positively about local policies and leg ulcer pathways which they worked within, informed by

For peer review only - http://bmjopen.bmj.com/site/about/guidelines.xhtml

NICE/SIGN. These were described as useful by interviewees and they talked about referring to them if they were stuck about where to go next with a patient. They were thought to be particularly useful for less experienced nurses. Interviewees articulated conviction that compression was the treatment of choice for someone with a VLU (despite the possibility that some nurses may not have the skills to put it into practice):

> *'I think we are very, sort of, strong in the terms that we are very sure…I feel like we, sort of, give quite a lot of confidence to that treatment. Like, we're just very sure that that is the treatment….But I think that helps that we are very clear that we know what the answer is. And the answer is compression.'* (S004).

DISCUSSION

Unwarranted variation in clinical practices across wound care services has been highlighted previously.[11] This study sought to understand how and why sub-optimal care happens. In doing so we have used the TDF[14 15] to explore influences on the provision of compression therapy for people with VLUs in the UK. Our analysis suggests that sub-optimal compression use is not necessarily due to lack of knowledge of appropriate care pathways, as has been described elsewhere.[23] Instead healthcare staff described a number of factors which they perceived made the provision of compression therapy more challenging. These can broadly be divided into two groups: organisational barriers (TDF domains environmental context and resources, knowledge, skills, behavioural regulation); and patient engagement barriers (TDF domains goals, beliefs about consequences).

Organisational barriers

Our findings echo those of other studies (e.g.[24]) which have suggested that the workload of community nursing services can impact on whether and how compression therapy is delivered. Rising patient caseloads are presenting growing challenges in community services,[25] due to both an ageing population and to significant decreases in the number of community nurses.[26] In this study, heavy workloads were seen as contributing to delays in initial leg and wound assessment and in monitoring progress. Work pressures could result in patients being attended by nurses without the necessary knowledge and skills to provide and monitor compression; a decline in the number of qualified District Nurses (holders of the Specialist Practitioner Qualification in District Nursing) in community nursing services[27] could be a contributory factor. Pressured workloads contributed to a lack of continuity of care too; perceived as problematic by nurses in this study as well as being a concern of patients who were interviewed about their experience of using compression therapy (Perry C, Atkinson R, Griffiths J, *et al*., 2021, unpublished).

For peer review only - http://bmjopen.bmj.com/site/about/guidelines.xhtml

Problems related to the knowledge and skills of healthcare practitioners in leg ulcer care and compression therapy[23 24] and the provision of adequate training[28] have been described. Traditionally, leg ulcer care has been provided through community nursing services. Interviewees in this study generally considered they had the knowledge and skills to manage venous leg ulcers themselves, but they identified lack of skills in other community staff, and lack of access to training. The main issue described was the time to attend training, alongside high staff turnover resulting in a constant need, and it was suggested that training provided 'on site' might help. Leg ulcer clinics where community nurses were encouraged to 'shadow' specialist leg ulcer nurses as a form of informal training, which could be fitted around their workload, were valued highly.

In this study, community nursing interviewees perceived the absence of specifically commissioned community-based specialist leg ulcer services, to which they could send patients, as a barrier to providing optimal leg ulcer care. Although referral to a specialist service may be necessary for complex leg ulcers, the majority of ulcers could arguably be managed in general community services.[29] Rather than referring to a specialist service, it may be that if issues relating to delivery models, workloads, skills and training in the community were addressed, appropriate care for patients with venous leg ulcers would be enabled. The NHSE-funded National Wound Care Strategy Programme is supporting a number of sites in the implementation of its lower limb wound recommendations to explore service delivery models and their potential for success.[8]

Organisational barriers to the provision of compression therapy related to prescribing were described. There were variations between bandaging systems available in different geographical areas which meant that not all compression therapies were available in all areas. Local restrictions on the types of compression systems available have been noted in other studies.[30] This could lead to difficulties for clinic or hospital-based nurses, whose patients came from different geographical areas, as they needed to be aware of different availabilities and prescribe accordingly, leading to a 'postcode lottery' of compression therapies. Including commissioners in discussions about service provision and feeding back about the impact of commissioning decisions may be useful in this situation.

Issues with prescribing were also noted when nurses were not qualified to prescribe compression therapies for their patients, instead having to request a prescription from a patient's General Practitioner. This could lead to delays in patients starting compression therapy or a temporary break in their treatment regimen, and interviews with patients found

For peer review only - http://bmjopen.bmj.com/site/about/guidelines.xhtml

evidence of patients not being in compression because they were waiting for prescribed hosiery to arrive (Perry C, Atkinson R, Griffiths J, *et al*., 2021, unpublished).

Patient engagement barriers

A good relationship between patient and health professional is an important foundation for the successful use of compression therapy.[31-33] In our study, nurses spoke of the importance of getting patients 'on board' with their compression therapy, and of supporting them in using it. Explanations were seen as key and it was almost universally thought that a patient who understood the aetiology of their VLU and the mechanism of compression therapy was more likely to continue to adhere to treatment. However it was evident in a study of patients (Perry C, Atkinson R, Griffiths J, *et al*., 2021, unpublished) that there was variation in patients' perceived understanding of the cause of their VLU and that many people had difficulty understanding this topic. There was not a clear relationship between knowledge/understanding and compression use: individuals who did and did not have an understanding of VLU aetiology or how compression worked, were adherent. It is possible that for some individuals the important thing was that explanations were offered: they were happy to rely on the recommendation of their nurse. This highlights the importance of the patient/health professional relationship.

Keeping patients 'on board' with their treatment was made harder because of the difficulties in explaining how long a VLU may take to heal and in supporting patients if their VLU become worse rather than improving. Patient interviews (Perry C, Atkinson R, Griffiths J, *et al*., 2021, unpublished) confirm that patients wanted to know how long healing might take and became very disheartened if their VLU became worse, thus underlining the importance of these issues being handled carefully by nurses. Encouraging some degree of 'self-management' or perhaps more accurately 'self-care' was thought to encourage continued compression use, although there is limited evidence of the benefits or risks of this in relation to compression therapy.

Intervention for change

More data were coded to organisational barriers in this study (TDF domains environmental context and resources, knowledge, skills, behavioural regulation) than to patient engagement barriers (TDF domains goals, beliefs about consequences). This could suggest that a multicomponent intervention will be required to support further delivery of compression in the treatment of VLUs. We recognise that rising patient caseloads and related workload pressures will present significant challenges to any attempt to enhance existing leg ulcer

For peer review only - http://bmjopen.bmj.com/site/about/guidelines.xhtml

services. Regardless, the organisational barriers highlighted in this study are similar to those highlighted by previous research,[11] but crucially this also suggests that there are similar opportunities to develop facilitated approaches to enable community nursing teams to make changes to practice and enhance the quality of patient care delivered.[25] This information can therefore be used to inform future improvement efforts locally.

Our analysis also suggests that there may be traction in addressing the barriers highlighted in relation to promoting appropriate compression use in people with VLUs. To this end, we have been working with one of the health care provider organisations involved in this work to produce an information leaflet which can be used by all staff to explain and promote the use of compression therapy. Strategies to encourage 'self-management' or 'self-care' would also merit further investigation.

Limitations

Although staff from the three participating community services were included in the sample, the majority came from one service. However, the issues raised by staff from all three services were similar. The research was conducted during the COVID-19 pandemic. All participants were therefore interviewed over the telephone. This may mean that our study participants were those who were naturally more comfortable with and experienced in talking over the telephone.

There were some challenges in using the TDF, as reported by other users.[8] Some data could be coded to different domains, decisions about coding in these instances were made through discussion between the research team. More data were coded into the environmental context and resources domain than any other. As anything that a healthcare professional does could be viewed as dependent on context,[14] the coding to this domain was examined carefully to ensure that it was the best fit. In one instance coding to the TDF domains split up data (on prescribing) that could usefully be considered together. However, this could be put back together in considering the implications of the findings.

CONCLUSIONS

We have presented an exploration of the factors that prevent or promote the appropriate use of compression therapy for people with VLUs, from the perspective of nurses involved in their care. Whilst patient engagement barriers may be easier and quicker to address than some of the organisational barriers identified, unless organisational barriers are also attended to it would seem unlikely that all people who would benefit from compression therapy will receive it.

For peer review only - http://bmjopen.bmj.com/site/about/guidelines.xhtml

Acknowledgements

The authors would like to express thanks to Cheryl Lenney (Chief Nurse, Manchester University NHS Foundation Trust), Alex Barker (Head of Nursing, Manchester Local Care Organisation) who were members of the Project Management Group (PMG). Thank you to our local Principal Investigators, Louise O'Connor (Lead Nurse for Tissue Viability and Infection Prevention, Manchester University NHS Foundation Trust, who was also a member of the PMG), Joanne Taylor (Tissue Viability Service Lead, Rochdale Care Organisation) and Martin Sylvester (Research Nurse, Mid Yorkshire Hospitals NHS Trust). We also thank our patient representative on the PMG, who has contributed to the work from the outset, including development of the protocol through to interpretation of the findings. The University of Manchester Wounds Research Patient and Public Involvement (PPI) Forum made useful comments on the interview schedule. We thank Francisco Espinoza for setting up the online participant registration form, and the nurse managers at participating Trusts who facilitated recruitment of their staff members as participants. Finally we would like to thank all of the staff who so generously gave their time to participate in this research during a very difficult period for NHS services.

Author contributions

NC conceived the idea and design for the overall project. RAA, JCD, JG, JFL, and PMW contributed to further development of the study design. CP collected the data. CP, RAA, JG and PMW conducted the data analysis. CP created the original draft of the manuscript. All authors contributed to the interpretation of study findings, critical revision of the manuscript for important intellectual content and approval of the final manuscript.

Competing interests None declared.

Funding

This work was supported by National Institute for Health Research (NIHR) Research for Patient Benefit (RfPB) scheme, grant number PB-PG-0817-20002.

Data sharing statement

Requests for access to data should be addressed to the corresponding author.

REFERENCES

1. NICE. *Varicose veins: diagnosis and management. Clinical guideline.* London: National Institute for Health and Care excellence, 2013.

For peer review only - http://bmjopen.bmj.com/site/about/guidelines.xhtml

2. Herber OR, Schnepp W, Rieger MA. A systematic review on the impact of leg ulceration on patients' quality of life. *Health Qual Life Outcomes* 2007;5:44.

3. Green J, Jester R. Health related quality of life and chronic venous leg ulceration: part 1. *Br J Community Nurs* 2009;14:S12-S17. doi: 10.12968/bjcn.2009.14.Sup6.45538

4. Green J, Jester R. Health related quality of life and chronic venous leg ulceration: part 2. Br J Community Nurs 2010;15:S12-S17. doi: 10.12968/bjcn.2010.15.Sup1.46906

5. Cullum N, Buckley H, Dumville J, *et al.* Wounds research for patient benefit: a 5-year programme of research. *Programme Grants for Applied Research 2016;*4:1-304.

6. O'Meara S, Cullum N, Nelson EA, *et al.* Compression for venous leg ulcers. *Cochrane Database Syst Rev* 2012;11:CD000265.

7. SIGN. *Management of chronic venous leg ulcers*: *A national clinical guideline (120):* Scottish Intercollegiate Guidelines Network, 2010.

8. National Wound Care Strategy Programme. *Improving Wound Care.* Accessed 12/01/22 https://www.nationalwoundcarestrategy.net/improving-wound-care/

9. Ashby RL, Gabe R, Ali S, *et al.* VenUS IV (Venous leg Ulcer Study IV) - compression hosiery compared with compression bandaging in the treatment of venous leg ulcers: a randomised controlled trial, mixed-treatment comparison and decision-analytic model. *Health Technol Assess* 2014;18:1-294.

10. Nelson EA, Bell-Syer SEM. Compression for preventing recurrence of venous ulcers. *Cochrane Database Syst Rev* 2012;8:CD002303.

11. Gray TA, Rhodes S, Atkinson RA, *et al.* (2018). Opportunities for better value wound care: a multiservice, cross-sectional survey of complex wounds and their care in a UK community population. *BMJ Open* 2018;8:e019440. doi:10.1136/bmjopen-2018-024859

For peer review only

For peer review only - http://bmjopen.bmj.com/site/about/guidelines.xhtml

12. Phillips P, Lumley E, Duncan R, *et al.* A systematic review of qualitative research into people's experiences of living with leg ulcers. *J Adv Nurs* 2018;74:550–563. doi: 10.1111/jan.13465

13. Weller CD, Buchbinder R, Johnston RV. Interventions for helping people adhere to compression treatments for venous leg ulceration. *Cochrane Database Syst Rev* 2016;3:CD008378.

14. Atkins L, Francis J, Islam R, *et al.* A guide to using the Theoretical Domains Framework of behaviour change to investigate implementation problems. *Implement Sci* 2017;12:77. doi: 10.1186/s13012-017-0605-9

15. Cane J, O'Connor D, Michie S. Validation of the theoretical domains framework for use in behaviour change and implementation research. *Implement Sci* 2012;7:37.

16. Francis JJ, Johnston M, Robertson C, *et al.* What is an adequate sample size? Operationalising data saturation for theory-based interview studies. *Psychol Health* 2010;25:1229-45.

17. Denscombe M. The good research guide for small scale social research projects. Philadelphia: Open University Press 2003:(2nd ed).

18. Bryman A. Social research methods. Oxford United Kingdom: Oxford University Press 2008: (3rd ed).

19. Britten N. Qualitative research. Qualitative interviews in medical research. *BMJ* 1995;311:251-253.

20. Gibbs G. Analyzing qualitative data. London United Kingdom: Sage 2007.

21. Ritchie J, Spencer L, O'Connor W. (2003). Carrying out qualitative analysis. In: Ritchie J, Lewis J, eds. Qualitative research in practice: A guide for social science students and researchers. CA: Sage 2003:220-262.

22. Tobin GA, Begley CM. Methodological rigour within a qualitative framework. *J Adv Nurs* 2004;48:388–396.

For peer review only - http://bmjopen.bmj.com/site/about/guidelines.xhtml

23. Weller CD, Richards C, Turnour AM, *et al.* Barriers and enablers to the use of venous leg ulcer clinical practice guidelines in Australian primary care: A qualitative study using the theoretical domains framework. *Int J Nurs Stud* 2019;103.

24. Franks PJ, Barker J, Collier M, *et al.* Management of patients with venous leg ulcers. Challenges and current best practice. *J Wound Care* 2016;25:s1-s67.

25. Swift A, Punshon G. *District Nursing Today. The view of District Nurse Team Leaders in the UK.* London: The Queen's Nursing Institute, International Community Nursing Observatory, 2019.

26. Nuffield Trust. *The NHS workforce in numbers. Facts on staffing and staff shortages in England.* Accessed 09/11/21www.nuffieldtrust.org.uk/resource/the-nhs-workforce-in-numbers

27. QNI. *Report on District Nurse Education in the United Kingdom 2017 – 2018.* London: The Queen's Nursing Institute, 2019.

28. CLAHRC GM. *ILUMIN: The Leg Ulcer Quality Improvement Programme Summary Report.* Collaboration for Leadership in Applied Health Research and Care Greater Manchester, 2019.

29. NICE. *Overview of management of a person with a venous leg ulcer.* Accessed 30/11/21https://cks.nice.org.uk/topics/leg-ulcer-venous/management/venous-leg-ulcers/#when-to-refer

30. Oates O, Adderley U. Survey of registered nurses' selection of compression systems for the treatment of venous leg ulcers in the UK. *J Tissue Viability* 2019;28:115–119. doi.org/10.1016/j.jtv.2019.02.004

31. Annells M, Macpherson Smith H, O'Neill J, *et al.* Compression bandaging for venous leg ulcers: the essentialness of a willing patient. *J Clin Nurs* 2008;17:350-359. doi: 10.1111/j.1365-2702.2007.01996.x

32. Douglas V. Living with a chronic leg ulcer: an insight into patients' experiences. *J Wound Care* 2001;10:355-360.

For peer review only - http://bmjopen.bmj.com/site/about/guidelines.xhtml

33. Van Hecke A, Verhaeghe S, Grypdonck M, *et al.* Processes underlying adherence to leg ulcer treatment: A qualitative field study. *Int J Nurs Stud* 2011;48:145-155.

For peer review only

Barriers and facilitators to compression therapy for people with venous leg ulcers – staff interview schedule

As part of this project we're really interested in understanding the barriers and facilitators to the delivery of compression therapy for people with venous leg ulcers, including factors that may operate at an individual, team and organisation level. We are also interested in your perspectives on how the behaviours of patients themselves might impact positively or negatively on compression use.

Question	Prompts	Rationale/TDF domains
**Can you tell me a little bit about your current role and your main responsibilities?**	• *How long have you been in this role?*<br>• *What did you do before?*<br>• *How is your team/clinic set up and organised?*	Easy first question, scene setting, supplements demographic information supplied, may reveal something important in relation to role which can be probed later (e.g. 'go to' leg ulcer person).
**From your perspective, is there a 'typical' leg ulcer patient? Could you describe them to me?**	• *Age/sex?*<br>• *Co-morbidities?*<br>• *Lifestyle factors?*	Scene setting.
**Can you tell me what 'gold standard' care and treatment for a patient with a venous leg ulcer looks like?**	• *Vascular assessment.*<br>• *Cleansing and debridement.*<br>• *Dressings and topical treatments.*<br>• *Compression therapy.*<br>• *Systemic therapy.*<br>• *Provision of care.*	Establishing something about interviewees' knowledge/experience – the background against which they practice.<br><br>TDF: knowledge; professional role and identity.
**What guides your choices about venous leg ulcer care?**	• *Knowledge and skills – training, peers, experience, preferences?*<br>• *Local policies?*<br>• *Research evidence - SIGN guidelines, Cochrane reviews, reading journals, online searches, specialist peers?*<br>• *Patient and carers' influence – lifestyle, preference, adherence.*	TDF: knowledge; skills; professional role and identity; beliefs about capabilities; optimism; beliefs about consequences; reinforcement; intentions; memory, attention and

For peer review only - http://bmjopen.bmj.com/site/about/guidelines.xhtml

Question	Prompts	Rationale/TDF domains
		decision processes; environmental context and resources; social influences; emotion; behavioural regulation.
**What is your experience of offering and delivering compression therapy to patients with venous leg ulcers?**	• *How important do you think compression is?*<br>• *What guides your decision to offer compression? What are your thought processes?*<br>• *How do you explain it to patients and what information do you routinely give them (any patient information/leaflets given)?*<br>• *Do you think patients understand the importance of it?*<br>• *Is there a particular type of compression you like?*<br>• *Do you start on full compression and reduce if patient cannot tolerate/start low and work up to full? Why?*<br>• *How easy or difficult do you find it to deliver compression therapy?*<br>• *What do you think are the necessary skills for effective compression delivery?*<br>• *Have you received relevant training to deliver compression therapy?*<br>• *Are there times when you would choose not to offer or deliver compression (even if it was clinically indicated)?*	TDF: knowledge; skills; professional role and identity; beliefs about capabilities; optimism; beliefs about consequences; reinforcement; intentions; goals; memory, attention and decision processes; environmental context and resources; social influences; emotion; behavioural regulation.
**I'd like to ask you specifically about what you see as the barriers to offering and delivering compression therapy, and what the facilitators are.**<br><br>**Are barriers and facilitators to offering compression therapy different to those of delivering compression therapy (may want to address separately if so)?**	***Individual level***<br>• *Do you personally find it easy/difficult to offer and deliver compression therapy?*<br>• *How important do you think the ABPI measurement is?*<br>• *What do you do if you are looking after a patient who has not had an ABPI measurement taken?*<br>• *How confident are you in using a Doppler?*<br>• *How confident are you in offering and delivering compression therapy?*<br>• *Is there anything that would make you more confident?*	TDF: knowledge; skills; beliefs about capabilities; emotion.

For peer review only - http://bmjopen.bmj.com/site/about/guidelines.xhtml

Question	Prompts	Rationale/TDF domains
**It may be easier to look at barriers and facilitators in turn. Barriers? Facilitators?**	***Team level***<br>• *Would any other team members influence whether or not you offer or deliver compression therapy (who and how)?*<br>***Organisational level***<br>• *Are there aspects of the community/clinic environment which influence whether or not you are able to offer and deliver compression?*<br>• *Are there any time constraints that influence whether or not you are able to offer and deliver compression?*<br>• *Are there any competing tasks that influence whether or not you are able to offer and deliver compression?*<br>• *Are there any specific issues (e.g. staffing) that influence whether or not you are able to offer and deliver compression?*<br>*If yes to any of the above – how big an issue is this, how does this issue affect your decision making, do you use simpler techniques/interventions so that less experienced staff can then continue using them?*<br>• *What systems are in place to assist with compression use (how easy or difficult is it to deliver compression)?*<br>• *What availability is there of Dopplers; staff training; patient information leaflets?*	TDF: professional role and identity; reinforcement; social influences.<br>TDF: professional role and identity; reinforcement; intentions; environmental context and resources; social influences; behavioural regulation.
**How might the views of your patients affect how you offer and deliver compression?**	• *Does compression therapy influence your relationships with patients? (does it affect communication?)*<br>• *How do you respond when a patient refuses to wear compression/is non-adherent?*<br>• *Do your colleagues generally agree with you about this approach to patient non-adherence?*<br>• *Do you find that you change from full compression to reduced compression?*<br>• *What are your views about reduced compression?*<br>• *Do you find patients adhere to reduced compression?*<br>• *Does not being able to deliver compression cause you to be worried or concerned?*	TDF: beliefs about consequences; goals; environmental context and resources; social influences; emotion.

For peer review only

Question	Prompts	Rationale/TDF domains
**Have you any views on why patients do or do not adhere to their compression therapy?**	• *Patient discomfort?*<br>• *Patient lifestyle?*<br>• *Patient beliefs about illness and treatment?*<br>• *Influence of partner/family/friends?*<br>• *Do your colleagues generally agree with you about patient non-adherence?*	TDF: beliefs about consequences; goals; environmental context and resources; social influences; emotion.
**What do you perceive to be the consequences of offering and delivering compression therapy?**	• *Does it influence the outcomes?*<br>• *Does it have an impact on your clinical workload and patient routines?*<br>• *What happens if you are not able to deliver compression as you would like to? (to patients, to colleagues, to yourself? Short and long-term consequences?)*	TDF: beliefs about consequences; optimism; intentions; goals; social influences; emotion.
**What would help you to overcome any difficulties/challenges that you perceive in offering and delivering compression therapy?**	• Skills training in using a Doppler machine?<br>• Other skills training?<br>• The number of Dopplers available?<br>• Other equipment?<br>• *What would you personally have to do to increase the number of patients wearing compression? (in the ideal world).*<br>• *If you wanted to implement changes in your own practice to increase the number of patients wearing compression, what would be some of the ways to do this?*	TDF: knowledge; skills; beliefs about capabilities; optimism; reinforcement; memory, attention and decision processes; environmental context and resources; behavioural regulation.
**Is there anything else about your experience of offering and delivering compression therapy that I haven't asked you about that you think it is important to say?**		Tapping into knowledge/experience of individual.
**Is there anybody else that you can think of (individual/groups) who you think it might be useful for me to talk to as part of this study?**		Snowballing/tapping into knowledge/experience of individual.

For peer review only - http://bmjopen.bmj.com/site/about/guidelines.xhtml

COREQ (Consolidated criteria for Reporting Qualitative research) Checklist

Topic	Item No.	Guide Questions/Description	Reported on Page No.
**Domain 1: Research team and reflexivity**			
Personal characteristics			
Interviewer/facilitator	1	Which author/s conducted the interview or focus group?	4
Credentials	2	What were the researcher's credentials? E.g. PhD, MD	Supplementary material below
Occupation	3	What was their occupation at the time of the study?	Supplementary material below
Gender	4	Was the researcher male or female?	Supplementary material below
Experience and training	5	What experience or training did the researcher have?	Supplementary material below
Relationship with participants			
Relationship established	6	Was a relationship established prior to study commencement?	Supplementary material below
Participant knowledge of interviewer	7	What did the participants know about the researcher? E.g. personal goals, reasons for doing the research.	Supplementary material below
Interviewer characteristics	8	What characteristics were reported about the interviewer/facilitator? E.g. bias, assumptions, reasons and interests in the research topic.	Supplementary material below
**Domain 2: Study design**			
Theoretical framework			
Methodological orientation and theory	9	What methodological orientation was stated to underpin the study? E.g. grounded theory, discourse analysis, ethnography, phenomenology, content analysis.	4
Participant selection			
Sampling	10	How were participants selected? E.g. purposive, convenience, consecutive, snowball.	4
Method of approach	11	How were participants approached? E.g. face-to-face, telephone, mail, email.	4
Sample size	12	How many participants were in the study?	5
Non-participation	13	How many people refused to participate or dropped out? Reasons?	Supplementary material below
Setting			
Setting of data collection	14	Where was the data collected? E.g. home, clinic, workplace.	4
Presence of non-participants	15	Was anyone else present besides the participants and researchers?	N/A
Description of sample	16	What are the important characteristics of the sample? E.g. demographic data, date.	4 and 6-7

For peer review only

Topic	Item No.	Guide Questions/Description	Reported on Page No.
Data collection			
Interview guide	17	Were questions, prompts, guides provided by the authors? Was it pilot tested?	4 and Supplementary File 1
Repeat interviews	18	Were repeat interviews carried out? If yes, how many?	N/A
Audio/visual recording	19	Did the research use audio or visual recording to collect the data?	5
Field notes	20	Were field notes made during and/or after the interview or focus group?	Supplementary material below
Duration	21	What was the duration of the interviews or focus group?	5
Data saturation	22	Was data saturation discussed?	4
Transcripts returned	23	Were transcripts returned to participants for comment and/or correction?	Supplementary material below
**Domain 3: analysis and findings**			
Data analysis			
Number of data coders	24	How many data coders coded the data?	4
Description of the coding tree	25	Did the authors provide a description of the coding tree?	8
Derivation of themes	26	Were themes identified in advance or derived from the data?	4-5
Software	27	What software, if applicable, was used to manage the data?	4
Participant checking	28	Did participants provide feedback on the findings?	5
Reporting			
Quotations presented	29	Were participant quotations presented to illustrate the themes/findings? Was each quotation identified? E.g. participant number.	8 and throughout findings
Data and findings consistent	30	Was there consistency between the data presented and the findings?	Throughout findings
Clarity of major themes	31	Were major themes clearly presented in the findings?	Throughout findings
Clarity of minor themes	32	Is there a description of diverse cases or discussion of minor themes?	Throughout findings

Developed from: Tong A, Sainsbury P, Craig J. Consolidated criteria for reporting qualitative research (COREQ): a 32 item checklist for interviews and focus groups. *International Journal for Quality in Health Care.* 2007. Volume 19, Number 6: pp.349-357.

Supplementary material

Below is presented additional detail about issues that it was not possible to present in the main paper, arranged under the COREQ headings.

For peer review only

Research team and reflexivity
Please see page 4 of paper, with supplementary material below.

Personal characteristics
Interviews were conducted by one researcher (CP). She was female, held a PhD, and was an experienced interviewer who had received training in qualitative research methods over a number of years, including during the conduct of her PhD. At the time of the study the researcher was employed as a University Research Fellow.

Relationship with participants
There was no relationship between research participants and the researcher prior to the commencement of the study. Potential participants had been provided with details of the study by their manager and if they were interested in learning more had completed an on-line form. They were then contacted via email by the researcher. The researcher's role as a University researcher was explained to individual potential interviewees. It was ensured that researcher contact details were clearly available in case potential participants had further questions.

Study design
Please see main body of paper, pages 4-7, with supplementary material below.

Participant selection
Eighteen potential participants registered their interest in participating in the study; three of these people did not respond to interview invitations and so were not interviewed.

Data collection
We developed a semi-structured interview schedule (Supplementary File 1) with reference to the research aim, the literature, and the Theoretical Domains Framework. This schedule was 'sense checked' by practitioner members of the study team. The interview schedule was kept under review as the interviews progressed to ensure that we were generating relevant data. Interview participants were offered sight of transcripts, although nobody availed themselves of this. Field notes were used to provide context.

Analysis and findings
Please see main body of paper, pages 4-8 and throughout findings.

For peer review only

What promotes or prevents greater use of appropriate compression in people with venous leg ulcers? A qualitative interview study with nurses in the north of England using the Theoretical Domains Framework

Journal:	*BMJ Open*
Manuscript ID	bmjopen-2022-061834.R1
Article Type:	Original research
Date Submitted by the Author:	20-May-2022
Complete List of Authors:	Perry, Catherine ; The University of Manchester, Division of Population Health, Health Services Research and Primary Care, School of Health Sciences<br>Atkinson, Ross; The University of Manchester, Division of Nursing, Midwifery and Social Work, School of Health Sciences, Faculty of Biology, Medicine and Health<br>Griffiths, Jane; The University of Manchester, Division of Nursing, Midwifery and Social Work, School of Health Sciences<br>Wilson, Paul; University of Manchester, Division of Population Health, Health Services Research and Primary Care, School of Health Sciences<br>Lavallée, Jacqueline; The University of Manchester, Division of Medical Education, School of Medical Sciences<br>Mullings, Julie; Manchester University NHS Foundation Trust<br>Cullum, Nicky; The University of Manchester, School of Health Sciences; Manchester University NHS Foundation Trust, MAHSC<br>Dumville, Jo C.; The University of Manchester, Division of Nursing, Midwifery and Social Work, School of Health Sciences
**Primary Subject Heading**:	Nursing
Secondary Subject Heading:	Nursing, Qualitative research
Keywords:	Vascular medicine < INTERNAL MEDICINE, PRIMARY CARE, QUALITATIVE RESEARCH, WOUND MANAGEMENT

I, the Submitting Author has the right to grant and does grant on behalf of all authors of the Work (as defined in the below author licence), an exclusive licence and/or a non-exclusive licence for contributions from authors who are: i) UK Crown employees; ii) where BMJ has agreed a CC-BY licence shall apply, and/or iii) in accordance with the terms applicable for US Federal Government officers or employees acting as part of their official duties; on a worldwide, perpetual, irrevocable, royalty-free basis to BMJ Publishing Group Ltd ("BMJ") its licensees and where the relevant Journal is co-owned by BMJ to the co-owners of the Journal, to publish the Work in this journal and any other BMJ products and to exploit all rights, as set out in our licence.

The Submitting Author accepts and understands that any supply made under these terms is made by BMJ to the Submitting Author unless you are acting as an employee on behalf of your employer or a postgraduate student of an affiliated institution which is paying any applicable article publishing charge ("APC") for Open Access articles. Where the Submitting Author wishes to make the Work available on an Open Access basis (and intends to pay the relevant APC), the terms of reuse of such Open Access shall be governed by a Creative Commons licence – details of these licences and which Creative Commons licence will apply to this Work are set out in our licence referred to above.

Other than as permitted in any relevant BMJ Author's Self Archiving Policies, I confirm this Work has not been accepted for publication elsewhere, is not being considered for publication elsewhere and does not duplicate material already published. I confirm all authors consent to publication of this Work and authorise the granting of this licence.

For peer review only

What promotes or prevents greater use of appropriate compression in people with venous leg ulcers? A qualitative interview study with nurses in the north of England using the Theoretical Domains Framework

Authors

Catherine Perry, Division of Population Health, Health Services Research and Primary Care, School of Health Sciences, University of Manchester, UK.

(Corresponding author: Catherine.perry@manchester.ac.uk)

Ross A Atkinson, Division of Nursing, Midwifery and Social Work, School of Health Sciences, University of Manchester, UK.

Jane Griffiths, Division of Nursing, Midwifery and Social Work, School of Health Sciences, University of Manchester, UK.

Paul M Wilson, Division of Population Health, Health Services Research and Primary Care, School of Health Sciences, University of Manchester, UK.

Jacqueline F Lavallée, Division of Medical Education, School of Medical Sciences, University of Manchester, UK.

Julie Mullings, Manchester University NHS Foundation Trust, Manchester, UK.

Nicky Cullum, Division of Nursing, Midwifery and Social Work, School of Health Sciences, University of Manchester, and MAHSC, Manchester University NHS Foundation Trust, Manchester, UK.

Jo C Dumville, Division of Nursing, Midwifery and Social Work, School of Health Sciences, University of Manchester, UK.

Disclaimer

The views expressed in this article are those of the authors and not necessarily those of the NHS, National Institute for Health and Care Research, or the Department of Health and Social care.

For peer review only - http://bmjopen.bmj.com/site/about/guidelines.xhtml

Ethics approval

Ethical approval was received in 2019 from the London Bridge NHS Research Ethics Committee (Ref 19/LO/0826).

Patient consent for publication

Not required.

Word count

6165

For peer review only

For peer review only - http://bmjopen.bmj.com/site/about/guidelines.xhtml

What promotes or prevents greater use of appropriate compression in people with venous leg ulcers? A qualitative interview study with nurses in the north of England using the Theoretical Domains Framework

ABSTRACT

Objectives

To investigate factors that promote and prevent the use of compression therapy in people with venous leg ulcers.

Design

Qualitative interview study with nurses using the Theoretical Domains Framework.

Setting

Three NHS Trusts in England.

Participants

Purposive sample of 15 nurses delivering wound care.

Results

Nurses described factors which made provision of compression therapy challenging. Organisational barriers (TDF domains environmental context and resources/knowledge, skills/behavioural regulation) included heavy/increasing caseloads; lack of knowledge/skills and the provision of training; and issues related to prescribing (variations in bandaging systems/whether nurses could prescribe). Absence of specialist leg ulcer services to which they could refer was perceived as a barrier to providing optimal care by some community-based nurses. Compression use was thought to be promoted by clinics for timely initial assessment; continuity of staff and good liaison between vascular/leg ulcer clinics and community teams; clear local policies and care pathways; and opportunities for training such as 'shadowing' in vascular/leg ulcer clinics. Patient engagement barriers (TDF domains goals/beliefs about consequences) focussed on getting patients 'on board' with compression, and supporting them in using it. Clear explanations were seen as key in promoting compression use.

Conclusions

Rising workload pressures present significant challenges to enhancing leg ulcer services. There may be opportunities to develop facilitated approaches to enable community nursing teams to make changes to practice, enhancing quality of patient care. The majority of venous leg ulcers could be managed in the community without referral to specialist community services if issues relating to workloads/skills/training are addressed. Barriers to promoting compression use could also be targeted, for example through the development of clear patient information leaflets. Whilst patient engagement barriers may be easier/quicker

For peer review only - http://bmjopen.bmj.com/site/about/guidelines.xhtml

to address than organisational barriers, unless organisational barriers are addressed it seems unlikely that all people who would benefit from compression therapy will receive it.

Strengths and limitations of this study

- Using qualitative interviews with nurses delivering wound care enabled an in-depth exploration of what promotes or prevents the use of compression therapy in people with venous leg ulcers, from the perspective of staff.
- Initial inductive analysis of interview data ensured that all data were accounted for, and the use of the Theoretical Domains Framework provided a theoretical structure for understanding factors that promoted or prevented compression us.
- Participating nurses were from three Trusts (provider organisations) in the North of England, staff in other geographical regions may have other views.
- All interviews were conducted over the telephone due to the COVID-19 pandemic, so those less happy with using the telephone may not have participated.

INTRODUCTION

Background to this study

Venous leg ulcers (VLUs) are common, open wounds on the lower leg which take more than two weeks to heal.[1] Problems with the veins in the leg (such as damage to the valves, or blockages), or impaired action of the calf muscles, prevent optimal blood flow to the heart. This causes venous stasis, increasing the pressure in the veins and triggering a complex change in tissue physiology that can result in open wounds which heal slowly, if at all. Leg ulcers are more prevalent in older people. They can be painful, impair mobility and sleep, restrict work and leisure activities and reduce psychological well-being, impacting greatly on health-related quality of life.[2-4] Complete wound healing has been identified as the main outcome desired by people with VLUs.[5]

In the UK, VLU care is commonly delivered in the community, in patients' homes or clinics, by nurses or other health professionals. Whilst a wide range of largely nurse-delivered community-focused interventions are available to treat VLUs (bandages, hosiery, dressings and topical agents among others), only compression therapy is supported by a convincing level of evidence[6] and recommended by clinical guidelines.[7 8] Compression therapy, in the form of bandages or stockings, is cost-effective and has become the first-line, evidence-based treatment for VLUs.[6 9] In addition, compression hosiery is current standard practice for prevention.[10]

For peer review only - http://bmjopen.bmj.com/site/about/guidelines.xhtml

Compression works when used as intended, but delivery and adherence can be problematic. For example, previous research has highlighted unwarranted variation in clinical practices across wound care services in the north of England.[11] This included significant underuse of compression therapy where clinically appropriate. Understanding how and why sub-optimal delivery of evidence-based practices happens is an essential prerequisite to developing strategies to enhance the quality of care delivered. Whilst there is recognition that patient factors may play a role, including discomfort, patients' understanding of the causes of ulceration, and their beliefs about compression in terms of healing and symptom control,[12, 13] less is known about the influences on professional behaviours. The aim of this study was to explore, from the perspective of nurses delivering wound care, the factors that promote or prevent the use of compression therapy for people with VLUs.

Theoretical underpinning

Understanding existing professional practices with a view to promoting positive changes in individual and collective behaviour requires an understanding of the influences on behaviours in the context in which they occur.[14] The Theoretical Domains Framework (TDF) is a comprehensive, theory-informed approach for identifying determinants of professional behaviour (cognitive, affective, social and environmental).[11 12] Organised across 14 conceptual domains (see Table 1), the TDF has been applied across several healthcare settings to explore professional practices and behaviours.[14] We used the TDF to investigate the factors that promote and prevent the use of compression therapy as a treatment for VLUs; thus identifying potentially modifiable determinants of implementation and providing a theoretical foundation to link these to targets for behaviour change.

For peer review only - http://bmjopen.bmj.com/site/about/guidelines.xhtml

Table 1 The Theoretical Domains Framework: domains and definitions[15]

Domain	Definition
Knowledge	An awareness of the existence of something
Skills	An ability or proficiency acquired through practice
Social/professional role and identity	A coherent set of behaviours and displayed personal qualities of an individual in a social or work setting
Beliefs about capabilities	Acceptance of the truth, reality, or validity about an ability, talent or facility that a person can put to constructive use
Optimism	The confidence that things will happen for the best or that desired goals will be attained
Beliefs about consequences	Acceptance of the truth, reality or validity about the outcomes of a behaviour in a given situation
Reinforcement	Increasing the probability of a response by arranging a dependent relationship or contingency between the response and a given stimulus
Intentions	A conscious decision to perform a behaviour or a resolve to act in a certain way
Goals	Mental representations of outcomes and states that an individual wants to achieve
Memory, attention and decision processes	The ability to retain information, focus selectively on aspects of the environment and choose between two or more alternatives
Environmental context and resources	Any circumstance of a person's situation or environment that discourages or encourages the development of skills and abilities, independence, social competence and adaptive behaviour
Social influences	Those interpersonal processes that can cause individuals to change their thoughts, feelings or behaviours
Emotion	A complex reaction pattern, involving experiential, behavioural, and psychological elements, by which the individual attempts to deal with a personally significant matter or event
Behavioural regulation	Anything aimed at managing or changing objectively observed or measured actions

For peer review only

METHODS

Sample

We purposively sampled participants from three NHS Trusts in the North of England. All were qualified nurses providing leg ulcer care within the community, including community nurses and nurses who worked in hospital outpatient clinics.

Participant recruitment and data generation

Recruitment and data generation took place between February 2020 and January 2021. Potential participants were contacted by their manager, who provided them with a brief description of the study and a link to an online form to register their interest in participating. Those completing the online form were contacted via email, including a Participant Information Sheet, by a researcher. If they were happy to participate a date and time were arranged for interview. Three potential participants who completed the online form did not go on to interview. Sampling was continued until it was judged that data saturation had been reached.[16]

We had planned to conduct face-to-face interviews, however, because of COVID-19 restrictions, all interviews were conducted over the telephone. Verbal consent was taken and digitally audio-recorded.

We undertook semi-structured interviews as they offer a good way to generate data on individuals' experiences.[17] We developed a semi-structured interview schedule (Supplementary File 1) with reference to the research aim, the literature, and the TDF. Interview questions defined the area to be explored[18] but allowed the interviewer or interviewee to diverge in order to follow up particular areas in more detail.[19] With the permission of participants, interviews were digitally audio-recorded and transcribed.

Data analysis

We uploaded interview transcripts onto NVivo software to aid data management.[20] Initially, an inductive thematic analysis was undertaken to create a framework for the data.[21] This was followed by a deductive analysis in which the data from the framework were mapped on to the TDF domains

The initial inductive analysis was carried out by the lead qualitative researcher who had conducted all of the interviews (CP) and to ensure dependability[22] the developing analytical framework was shared and discussed with the research team. Similarly, the deductive analysis mapping specific barriers and enablers within each TDF domain was carried out by

For peer review only - http://bmjopen.bmj.com/site/about/guidelines.xhtml

one author (CP) in regular discussion with the research team, with additional sense-making and insight provided by the Study Steering Group. To ensure credibility[22] a near final version of the analysis was presented to a group of nurses experienced in wound care from across England, who agreed that the findings (and our interpretation of them) reflected their own experiences and/or made sense to them.

Patient and public involvement

A patient representative sits on the Study Steering Group and has been an active member of the team, contributing to the work from the writing of the study protocol through to discussion of emerging findings and their interpretation. The study was also presented to the University of Manchester Wounds Research Patient and Public Involvement (PPI) Forum. In particular members of the forum were consulted about interview schedule questions covering issues pertinent to them, for example questions regarding the importance of the staff-patient relationship.

RESULTS

Participant characteristics

We interviewed 15 nurses who delivered wound care, with interviews between 25 and 69 minutes in length. Interviewees were all female and comprised nine community nurses or managers, three vascular nurse specialists and three leg ulcer nurses, with a range of experience represented (Table 2).

For peer review only - http://bmjopen.bmj.com/site/about/guidelines.xhtml

Table 2 Participant characteristics

Participant ID<br><br>(cl clinic-based role, co community-based role)	Job title (self-reported)	Length of service as qualified nurse (years)	Time in current role (years)	Highest educational qualification	Training
001cl	Sister, Community Leg Ulcer Service	31-40	11-20	Diploma	N18 (compression therapy)<br>V150 (prescribing)
002co	Community Specialist Practitioner Student	6-10	6-10	Currently completing BSc in District Nursing	'In house' leg ulcer training
003cl	Vascular Leg Ulcer Nurse	21-30	0-5	BSc	Tissue viability and leg ulcer management modules (accredited)<br>Ongoing wound management, leg ulcer updates, courses, conferences.
004cl	Vascular Nurse Specialist	11-20	6-10	MSc	Leg ulcer management (University, stand-alone module)<br>Wound debridement (University, stand-alone module)<br>Attendance at Wounds UK Conferences
005co	Community Staff Nurse	11-20	6-10	Diploma in Nursing with advanced standing	Leg ulcer management Level 6<br>V150 (prescribing)<br>Online wound care training sessions
006co	Community Staff Nurse	21-30	11-20	BSc (hons) Nursing Studies	N18 leg ulcer course<br>Leg ulcer conferences including Lindsay Leg Club<br>Annual ‘in-house’ wound care training
007cl	Clinical Nurse Specialist (vascular)	31-40	11-20	Diploma in Nursing	Leg Ulcer management<br>Mentorship (supporting learning in practice) Community Practitioner

For peer review only - http://bmjopen.bmj.com/site/about/guidelines.xhtml

					Nurse Prescribing Vascular Disease Management (University)
008co	District Nursing Sister	6-10	6-10	Specialist Practitioner District Nursing	Leg Ulcer Management (University)<br>V150 Community Prescriber.
009co	Leg Ulcer Nurse	21-30	0-5	RGN	Leg Ulcer Management (online, University)
010cl	Leg Ulcer Nurse	11-20	0-5	Diploma in Nursing	Leg Ulcer management Masters module (online)<br>Wound UK courses<br>Lindsay leg Club course
011co	Practice Educator for Community Vascular Services	11-20	0-5	Advanced Diploma in adult Nursing	Leg Ulcer management Masters module (online)<br>Nurse Prescriber for Community Formulary<br>Trust 2 day wound care course,<br>Regular TVN and VNS wound care updates
012co	Senior Community Nurse	0-5	0-5	BA Nursing	Leg Ulcer Course (1 day) run by Urgo<br>'In house' courses<br>Nurse Prescriber
013co	Community Nurse	0-5	0-5	BSc (hons) Nursing	Trust training - wound care and compression
014co	Neighbourhood Lead	11-20	11-20	Masters (Research)	Numerous training courses over years
015co	Community Nurse	6-10	6-10	RGN	Leg ulcer module (10 days)<br>Drug Company study days

BA Bachelor of Arts BSc Bachelor of Science MSc Master of Science RGN Registered General Nurse TVN Tissue Viability Nurse

VNS Vascular Nurse Specialist

For peer review only - http://bmjopen.bmj.com/site/about/guidelines.xhtml

Relevant domains and key themes

We coded data to seven TDF domains: environmental context and resources, goals, knowledge, skills, beliefs about consequences, behavioural regulation and intentions. The domains of knowledge and skills are presented together as they were linked in the data by the issue of training. We did not have any data that related to the other TDF domains. We did not expect to have data in all of the domains due to the comprehensive nature of the framework. Domains and their associated themes are displayed in Table 3. The findings are presented in relation to the TDF domains, with the domains to which most data were coded presented first. Quotations from participants are used to illustrate points made, and are identified with a participant ID and indication of whether they are primarily community-based (co) or clinic-based (cl).

Table 3 Domains and associated themes

Domain	Theme
Environmental context and resources	Lack of commissioned leg ulcer services<br>Delays in initial leg and wound assessment<br>Lack of continuity of care<br>'Joined up' services and multidisciplinary teams<br>Prescribing issues<br>Application of compression as physically hard work
Goals	Getting patients 'on board'<br>Explaining timeframes around healing
Knowledge and skills	Gaps in knowledge and skills<br>Training
Beliefs about consequences	Encouraging compression use<br>Response to non-adherence
Behavioural regulation	Variations in bandaging systems used
Intentions	Local policies and leg ulcer pathways<br>Compression as treatment of choice

Environmental context and resources

Lack of commissioned leg ulcer services

In some areas the lack of specifically commissioned leg ulcer services was mentioned as a barrier to the provision of appropriate compression therapy; in these areas leg ulcer care was provided as part of general community nursing services: *'It's just wrapped up in district*

For peer review only - http://bmjopen.bmj.com/site/about/guidelines.xhtml

nursing.' (S014co). Community nurses discussed the challenge of offering optimal care, either because of perceived lack of skills in compression therapy or because of other responsibilities, resulting in apparently eligible people not receiving compression: *'You're not able to follow up….and like offer the treatment as they should anyway because of all the other things you have to worry about.'* (S002co). Non-housebound patients were not eligible for home visits from community nursing services; in some areas they could attend community nurse-led clinics but in others they received leg ulcer care from practice nurses at their GP surgery, and these nurses were perceived as less likely to be trained in compression therapy: *'Sometimes the practice nurses are not competent to do the compression bandaging.'* (S004cl). These issues could affect the type of compression a patient was offered.

In areas with commissioned leg ulcer services and leg ulcer clinics, the clinics were highly thought of and seen as facilitating good quality care, incorporating appropriately trained staff and continuity:

> *'The leg ulcer clinics in [name of areas] are fabulous. Fabulous for patients, great for continuity, but also, great for anybody who has joined the district nursing team to make sure we've got that consistency across the* [area].*'* (S014co).

Questions were, however, raised about the accessibility of some clinics, and it was suggested that patients may have difficulty getting to clinic locations on multiple/frequent occasions.

Delays in initial leg and wound assessment

The importance of fast, timely, initial assessment of someone with a suspected VLU was a universal theme: *'Getting the treatment in place quickly, because otherwise it can become….like the chronicity increases and the chance of healing decreases.'* (S004cl). However, delays in initial assessment were often described. This could be because community nurses did not recognise the need for specialised assessment of a wound: *'Nobody's thought about doing a Doppler. And that might be because they're not experienced, so hadn't thought about it.'* (S006co); or that they lacked the skills to carry it out: *'There's only a select few of us within the team who can complete the Doppler assessment…..there's just not enough of us.'* (S005co); (the Doppler assessment measures ankle-brachial pressure index (ABPI) and is an important component of initial assessment prior to compression use). Delays in access to Doppler were also ascribed to the general pressure of work in the community:

For peer review only - http://bmjopen.bmj.com/site/about/guidelines.xhtml

> *'So, pressure of work most definitely is a barrier……you could go in, see the patient…..you know put the dressing on. You are in and out of that house in 15 minutes, aren't you? You can also send your unregistered, or registered but non-nurse members of the team and so, even though our leg ulcer pathway, if you like, says, the gold standard, after two weeks you should be doing a Doppler, pressure of work, if you've got loads and loads of visits and not many staff and you've got low grades of staff, well then, that can slip can't it?'* (S014co).

Even in areas with dedicated leg ulcer clinics delays in getting an initial assessment were described:

> *'There needs to be more of them* [leg ulcer clinics]*, because patients are still waiting too long for assessment. So we see lots of patients in vascular* [clinic] *that have waited three, four months. So they've been seeing the district nurses, waiting three or four months to go and see the leg ulcer clinic, and in that time their leg's getting worse and worse and worse, and in the end, the GP's referred them to Vascular. So they've ended up in secondary care, because the waiting lists in primary care are too long.'* (S003cl).

Two interviewees highlighted the value of specific assessment clinics which had been established to enable timely assessment and one spoke about a new role that she had undertaken providing community vascular support, which she said could lead to more timely assessments. More broadly, interviewees spoke about promoting awareness of leg care. One talked about *'a Legs Matter week….what they tend to do is promote healthy legs and promote compression and promote good skincare*' (S006co) and another suggested *'it's not just patients, just nurses in general need [awareness raising]'* (S001cl).

Lack of continuity of care

A lack of continuity of nursing care, with different staff attending patients, was perceived as a barrier to effective compression therapy. It was more a problem for community teams than in leg ulcer clinics, and could result in staff not having an overview of a patient's care, knowledge of their disease or treatment history, or reasoning behind clinical decisions. A nurse explained:

> *'The teams are so large, what will happen is, somebody will start the reduced compression. And then obviously, a different person goes in next time, and they will think they're in reduced compression, because they've got a problem with their arterial line. And no one will ever increase that compression, because they assume, because they're in reduced compression, that they've got some, you know…'* (S003cl).

This situation could be exacerbated by the lack of availability of electronic patient records in the community. A vascular clinic nurse commented:

For peer review only - http://bmjopen.bmj.com/site/about/guidelines.xhtml

> *'So that's another thing that adds to it. When everyone's on different [patient record systems]. "We can't access the [electronic] hospital records". I don't think the district nurses have got access. We have but they don't.'* (S010cl).

Lack of continuity in staffing was perceived as leading to a situation where *'somebody else thinks somebody else's, you know, it's somebody else's responsibility'* (S006co). One interviewee described a change in working practices, 'zonal working' where a smaller group of nurses covered a defined geographical area, as increasing the likelihood of staff continuity. In addition, patients having contact details for the nursing team, for example through a telephone help-line, was thought to enhance continuity of care and communication between staff and patients.

'Joined up' services and multidisciplinary teams

A lack of communication between different services was identified as a barrier to optimal care. A patient with a VLU could be seen by hospital vascular services, leg ulcer clinics and district nursing services, as well as have contact with primary and secondary care services for issues other than their VLU. It was reported that these services did not necessarily communicate well with each other, resulting in unnecessary duplication (e.g., where a patient gets referred to *both* a leg ulcer clinic and a vascular clinic) or unnecessary referral (e.g., where a patient had undergone investigations whilst a hospital inpatient but the results of these investigations were not communicated to the other services). Patients not receiving their compression therapy whilst hospital inpatients was frequently mentioned:

> *'Another barrier to our work is that we can be working really hard with a patient, they become unwell, they're admitted to hospital for something completely different. And the first thing they do is take the compression bandages off their legs and just stick a plaster on. Because nobody within hospital can do that treatment either.'* (S001cl).

Conversely, good liaison between vascular or leg ulcer clinics and community nursing teams was described as a facilitator of good care. Often a patient would be seen in a vascular or leg ulcer clinic, a treatment plan drawn up and they would be returned to community teams to continue the treatment. The majority of both clinic-based nurses and community-based nurses reported good communication and liaison between the two: *'Any of the patients now that come to see me leave this clinic with a concise treatment plan…..I've got good established links with community'* (S003cl); *'We have the numbers of all the vascular specialist nurses, so if we feel like we just need to ask a little bit of advice… they'll get back to us straight away'* (S013co).

For peer review only - http://bmjopen.bmj.com/site/about/guidelines.xhtml

Multidisciplinary teams, including nurses, physiotherapists, podiatrists and social workers working well together were described as facilitating high quality VLU care and compression therapy. The importance of working with the care system was also highlighted in terms of care workers being able to support patients with their compression therapy. One nurse also suggested working with care workers on training:

> *'The care providers, because they're often quite low-paid, so they have minimal training and that's why we're, our vision is joint training. Because, if they could come on our leg ulcer morning or updates, so that they understand the importance of the compression.'* (S014co).

Prescribing issues

Compression therapy is a prescribed treatment. Nurses who had been trained as independent prescribers were able to prescribe compression, others had to ask a patient's GP to prescribe the treatment. This could cause delays and could result in patients not having prescribed treatment in time for their next nurse appointment:

> *'[Patient takes] your form to the GP. It has to sit at the GP's for 48 hours. They then do a prescription. The patient has to pick the prescription up, take it to the chemist. You might wait another 48 hours to pick your prescription up.'* (S001cl).

Most clinic nurses and some community nurses had stocks of compression which they could use if necessary (patients were often started off in their compression from these stocks), but this was not always the case.

Application of compression as physically hard work

Four interviewees reported that application of compression therapy, in some circumstances, can be physically hard work, particularly where the application takes place in patients' homes without any aids including treatment couches at the correct height. One interviewee commented: *'for district nurses it will often be on the floor on their hands and knees….so it is physically a little bit tricky'* (S004cl) and another: *'we've had a couple of staff that had got either bad backs or bad knees. So that takes them out of being able to do it for a couple of weeks'* (S012co). Suggested facilitators were working in pairs, and spreading out compression visits over several days in order to avoid having too many on one day, but these solutions often depended on adequate staffing.

Goals

Getting patients 'on board'

For peer review only - http://bmjopen.bmj.com/site/about/guidelines.xhtml

All Interviewees described the importance of getting patients 'on board' with their treatment, that is, in agreement with and involved with it, from the beginning: *'I think if they're not on board from the beginning, you're fighting a losing battle.'* (S005co). This could be difficult: *'Getting them on board to comply with the compression, and the correct compression. I think basically, that's the biggest one that we have a problem with.'* (S007cl). Explanations were seen as key to engaging patients: nurses were clear that if patients understood that their VLU was a symptom of an underlying long-term condition, and understood the mechanism of compression therapy, they were more likely to adhere to treatment.

In relation to explaining aetiology and compression therapy, interviewees talked about the availability and utility of written information to use with patients. A minority did not use written information at all and some thought that patients did not read information leaflets anyway. However, the majority of interviewees thought that written information could be useful:

> *'So it just gives them something to read at their own leisure, and then next time you go, they've got a bit more of an understanding of what you're talking about as well. So I find the leaflets do work quite well.'* (S008co).

Some interviewees had access to 'Trust leaflets' and product company leaflets were also mentioned, although worries were expressed about bias, which may be indicative of variation in the quality of this information. In order to be useful all interviewees thought that leaflets needed to have very simple, clear explanations, some said that pictures were helpful, and one person suggested alternative information formats such as short films.

In terms of getting patients 'on board', the majority of interviewees also spoke of giving patients options or choices, which might make them more likely to adhere to their compression therapy. One nurse explained:

> *'Like this is how I would feel you'd do best, however, these are your other options. And I'd rather give options at this point and formulate a plan, because you tend to get a little bit more concordance than saying well, this is what you are going to do.'* (S001cl).

There were a number of situations in which it could be possible to offer options, for example less obtrusive compression or being flexible with clinic appointments. All nurses agreed that it was important to build a relationship with their patient in which they could discuss treatment issues and work together towards the best outcome for the patient: '*A lot of it is getting them on board, building that relationship up with your patients…….and it's a journey together really.'* (S007cl); *'It's teamwork, it's not a one-way system.'* (S002co).

Explaining timeframes around healing

For peer review only - http://bmjopen.bmj.com/site/about/guidelines.xhtml

The difficulty in estimating how long a VLU may take to heal and explaining this to a patient could be a barrier to setting goals and the successful use of compression therapy. All interviewees said that patients always asked about how long healing would take: *'I think that's one of the first things they'll ask you.'* (S001cl). Nurses suggested that giving a specific time frame should be avoided: *'The worst thing you could do is give the time. Because, then they're going to sit and wait and say right it's been this long, and it's not healed.'* (S008co). A number of things that interviewees thought helped in explaining timeframes included being positive; talking about healing in small steps; describing what patients can do to speed up healing and issues that may slow healing; and being honest about ulcers that are likely to take a long time to heal, if at all.

One particular difficulty with trying to explain timeframes around healing was that in many cases nurses suggested healing of a VLU does not take a straightforward trajectory over time, and a wound may appear to get worse. Interviewees described patients becoming *'really disheartened'* (S005co). There was consensus that there was usually a discernible cause for an ulcer deteriorating, thus the important thing was to identify the cause and address it: *'You can try and look at a reason why it has happened, like infection. And then treat that cause…….and tell them* [patient] *that you've addressed that so there is some hope for them that will work.'* (S004cl).

Knowledge and skills

Gaps in knowledge and skills

There is some suggestion that not all community nurses have the necessary knowledge to recognise the need for a comprehensive leg and wound assessment, or the necessary skills to carry out this initial assessment (including ABPI). Interviewees also recognised a lack of skills among some community nurses in relation to the application of compression bandages: *'The next district nurse that's gone in is not competent in bandaging, so they've not been able to receive the treatment.'* (S003cl); and the monitoring of compression therapy:

> '[Patient] *who I sent back to the district nurses to look after…..nobody had bothered to put it* [compression bandages] *back on because she didn't really want it on. So nobody had gone through all the things again, why you need to have it, why it's going to help you….nobody had bothered putting it back on because she'd said "Oh I don't really want it".'* (S010cl).

Training

Interviewees identified issues with the provision of training to address the gaps in knowledge and skills as being a barrier to the provision of compression therapy. Although training

For peer review only - http://bmjopen.bmj.com/site/about/guidelines.xhtml

provision was generally considered to be adequate for those working in more specialised roles, for community nurses this was not always the case. A minority of interviewees thought that the availability of training was not good, and a majority mentioned access being more of an issue – it could be challenging to take time out to attend training because of workloads: *'The first thing that gets cancelled is training, unfortunately, 'cos obviously the needs of the patient come before training.'* (S005co). It was also noted that staff turnover in the community was high, so there was always a need for training. Some positive examples of training were given; a community nurse talked about training offered by her Tissue Viability Team which she described as *'really good….we do it yearly, so we do have leg ulcer training yearly and compression training'* (005co). Various facilitators of training were suggested, for example it was thought that provision of training 'on site' may get around issues of time to attend. Another interesting issue was the kind of 'informal' training that could be gained by nurses shadowing others in leg ulcer clinics, where these were available: *'You could go and spend an afternoon there and it's just that…..The experts are there, great.'* (S014co).

Beliefs about consequences

Encouraging compression use

All interviewees thought that frequent reiteration of explanations about compression being the best therapy was important in encouraging patients to continue using it. They talked about patients being able to 'self-manage' their care and compression therapy as a goal to be aimed for, which they perceived could contribute to the wider use of compression. However this *'can be difficult sometimes, but we're trying to encourage that self-caring as well and encourage them to take control over their care'* (S002co). One nurse suggested that barriers to self-care involved not only patients but the thinking and attitudes of nurses themselves:

> *'Self-management's quite a new…when I say new, I mean sort of, in the last few years. But it was always, sort of, the nurse came and did your dressing, and the nurse looked after it. And, I think, even for staff who've been around a long time it's hard getting that sort of, out of your head. So, it's about, sort of, changing your whole attitude and going in from the start and with the idea [that the patient will self-manage].'* (S006co).

Response to non-adherence

Many interviewees spoke about how their response to a patient who was not adhering to compression therapy was important in terms of whether that person might carry on, or would try compression again in the future: *'We have to ask why and is there anything else we can do to change that or make it better. Try and find the root of the cause of why they're saying no and then take it from there.'* (S002co). It was also universally agreed that there will always be patients who will not tolerate or adhere to compression therapy, and that this was

For peer review only - http://bmjopen.bmj.com/site/about/guidelines.xhtml

another point at which there was a need to be sensitive in responding in order to support the patient and keep the door open for them to try compression again in the future:

> *'I think if they are very sure they don't want to, and we do see…we do see a few like that, you just, I mean, you just kind of say that you understand their choice and, of course, it is their choice what they have on and we understand the reasons why. But that they have to also understand that, you know, healing will be delayed, or indeed will be very difficult to achieve without compression, in a nice way.'* (S004cl).

Behavioural regulation

Variation in available bandaging systems

Bandaging systems available for use varied geographically, as recommended by local Tissue Viability or Leg Ulcer Teams. When asked whether she was constrained in what compression therapy she could use for a patient based on where a patient lived and the bandage system used there one clinic-based nurse replied: *'I tend to because you just don't want to make life any more difficult'* (S003cl). Community nurses sometimes changed the compression type decided upon by specialist services because they are not able to provide it in their geographical area: *'We change them to* [name of compression brand] *because that's what we can…..that's what our ordering system allows us to order'* (S012co).

Intentions

We did not map any barriers to this domain. Instead, the majority of interviewees talked positively about local policies and leg ulcer pathways which they worked within, informed by NICE/SIGN. These were described as useful by interviewees and they talked about referring to them if they were stuck about where to go next with a patient. They were thought to be particularly useful for less experienced nurses. Interviewees articulated conviction that compression was the treatment of choice for someone with a VLU (despite the possibility that some nurses may not have the skills to put it into practice):

> *'I think we are very, sort of, strong in the terms that we are very sure…I feel like we, sort of, give quite a lot of confidence to that treatment. Like, we're just very sure that that is the treatment….But I think that helps that we are very clear that we know what the answer is. And the answer is compression.'* (S004cl).

DISCUSSION

Unwarranted variation in clinical practices across wound care services has been highlighted previously.[11] This study sought to understand how and why sub-optimal care happens. In doing so we have used the TDF[14 15] to explore influences on the provision of compression therapy for people with VLUs in the UK. Our analysis suggests that sub-optimal compression use is not necessarily due to lack of knowledge of appropriate care pathways, as has been

For peer review only - http://bmjopen.bmj.com/site/about/guidelines.xhtml

described elsewhere.[23] Instead healthcare staff described a number of factors which they perceived made the provision of compression therapy more challenging. These can broadly be divided into two groups: organisational barriers (TDF domains environmental context and resources, knowledge, skills, behavioural regulation); and patient engagement barriers (TDF domains goals, beliefs about consequences).

Organisational barriers

Our findings echo those of other studies (e.g.[24]) which have suggested that the workload of community nursing services can impact on whether and how compression therapy is delivered. Rising patient caseloads are presenting growing challenges in community services,[25] due to both an ageing population and to significant decreases in the number of community nurses.[26] In this study, heavy workloads were seen as contributing to delays in initial leg and wound assessment and in monitoring progress. Work pressures could result in patients being attended by nurses without the necessary knowledge and skills to provide and monitor compression; a decline in the number of qualified District Nurses (holders of the Specialist Practitioner Qualification in District Nursing) in community nursing services[27] could be a contributory factor. Pressured workloads contributed to a lack of continuity of care too; perceived as problematic by nurses in this study as well as being a concern of patients who were interviewed about their experience of using compression therapy (Perry C, Atkinson R, Griffiths J, *et al.*, 2021, unpublished).

Problems related to the knowledge and skills of healthcare practitioners in leg ulcer care and compression therapy[23 24] and the provision of adequate training[28] have been described. Traditionally, leg ulcer care has been provided through community nursing services. Interviewees in this study generally considered they had the knowledge and skills to manage venous leg ulcers themselves, but they identified lack of skills in other community staff, and lack of access to training. The main issue described was the time to attend training, alongside high staff turnover resulting in a constant need, and it was suggested that training provided ‘on site’ might help. Leg ulcer clinics where community nurses were encouraged to ‘shadow’ specialist leg ulcer nurses as a form of informal training, which could be fitted around their workload, were valued highly.

In this study, community nursing interviewees perceived the absence of specifically commissioned community-based specialist leg ulcer services, to which they could send patients, as a barrier to providing optimal leg ulcer care. Although referral to a specialist service may be necessary for complex leg ulcers, the majority of ulcers could arguably be managed in general community services.[29] Rather than referring to a specialist service, it

For peer review only - http://bmjopen.bmj.com/site/about/guidelines.xhtml

may be that if issues relating to delivery models, workloads, skills and training in the community were addressed, appropriate care for patients with venous leg ulcers would be enabled. The National Health Service England (NHSE)-funded National Wound Care Strategy Programme is supporting a number of sites in the implementation of its lower limb wound recommendations to explore service delivery models and their potential for success.[8]

Organisational barriers to the provision of compression therapy related to prescribing were described. There were variations between bandaging systems available in different geographical areas which meant that not all compression therapies were available in all areas. Local restrictions on the types of compression systems available have been noted in other studies.[30] This could lead to difficulties for clinic or hospital-based nurses, whose patients came from different geographical areas, as they needed to be aware of different availabilities and prescribe accordingly, leading to a 'postcode lottery' of compression therapies. Including commissioners in discussions about service provision and feeding back about the impact of commissioning decisions may be useful in this situation.

Issues with prescribing were also noted when nurses were not qualified to prescribe compression therapies for their patients, instead having to request a prescription from a patient's General Practitioner. This could lead to delays in patients starting compression therapy or a temporary break in their treatment regimen, and interviews with patients found evidence of patients not being in compression because they were waiting for prescribed hosiery to arrive (Perry C, Atkinson R, Griffiths J, *et al*., 2021, unpublished).

Patient engagement barriers

A good relationship between patient and health professional is an important foundation for the successful use of compression therapy.[31-33] In our study, nurses spoke of the importance of getting patients 'on board' with their compression therapy, and of supporting them in using it. Explanations were seen as key and it was almost universally thought that a patient who understood the aetiology of their VLU and the mechanism of compression therapy was more likely to continue to adhere to treatment. However it was evident in a study of patients (Perry C, Atkinson R, Griffiths J, *et al*., 2021, unpublished) that there was variation in patients' perceived understanding of the cause of their VLU and that many people had difficulty understanding this topic. There was not a clear relationship between knowledge/understanding and compression use: individuals who did and did not have an understanding of VLU aetiology or how compression worked, were adherent. It is possible that for some individuals the important thing was that explanations were offered: they were

For peer review only - http://bmjopen.bmj.com/site/about/guidelines.xhtml

happy to rely on the recommendation of their nurse. This highlights the importance of the patient/health professional relationship.

Keeping patients ‘on board’ with their treatment was made harder because of the difficulties in explaining how long a VLU may take to heal and in supporting patients if their VLU become worse rather than improving. Patient interviews (Perry C, Atkinson R, Griffiths J, *et al*., 2021, unpublished) confirm that patients wanted to know how long healing might take and became very disheartened if their VLU became worse, thus underlining the importance of these issues being handled carefully by nurses. Encouraging some degree of ‘self-management’ or perhaps more accurately ‘self-care’ was thought to encourage continued compression use, although there is limited evidence of the benefits or risks of this in relation to compression therapy.

Intervention for change

More data were coded to organisational barriers in this study (TDF domains environmental context and resources, knowledge, skills, behavioural regulation) than to patient engagement barriers (TDF domains goals, beliefs about consequences). This could suggest that a multicomponent intervention will be required to support further delivery of compression in the treatment of VLUs. We recognise that rising patient caseloads and related workload pressures will present significant challenges to any attempt to enhance existing leg ulcer services. Regardless, the organisational barriers highlighted in this study are similar to those highlighted by previous research,[11] but crucially this also suggests that there are similar opportunities to develop facilitated approaches to enable community nursing teams to make changes to practice and enhance the quality of patient care delivered.[25] This information can therefore be used to inform future improvement efforts locally.

Our analysis also suggests that there may be traction in addressing the barriers highlighted in relation to promoting appropriate compression use in people with VLUs. To this end, we have been working with one of the health care provider organisations involved in this work to produce an information leaflet which can be used by all staff to explain and promote the use of compression therapy. Strategies to encourage ‘self-management’ or ‘self-care’ would also merit further investigation.

Limitations

Although staff from the three participating community services were included in the sample, the majority came from one service. However, the issues raised by staff from all three

For peer review only - http://bmjopen.bmj.com/site/about/guidelines.xhtml

services were similar. The research was conducted during the COVID-19 pandemic. All participants were therefore interviewed over the telephone. This may mean that our study participants were those who were naturally more comfortable with and experienced in talking over the telephone.

There were some challenges in using the TDF, as reported by other users.[8] Some data could be coded to different domains, decisions about coding in these instances were made through discussion between the research team. More data were coded into the environmental context and resources domain than any other. As anything that a healthcare professional does could be viewed as dependent on context,[14] the coding to this domain was examined carefully to ensure that it was the best fit. In one instance coding to the TDF domains split up data (on prescribing) that could usefully be considered together. However, this could be put back together in considering the implications of the findings.

CONCLUSIONS

We have presented an exploration of the factors that prevent or promote the appropriate use of compression therapy for people with VLUs, from the perspective of nurses involved in their care. Whilst patient engagement barriers may be easier and quicker to address than some of the organisational barriers identified, unless organisational barriers are also attended to it would seem unlikely that all people who would benefit from compression therapy will receive it.

Acknowledgments

The authors would like to express thanks to Cheryl Lenney (Chief Nurse, Manchester University NHS Foundation Trust), and Alex Barker (Head of Nursing, Manchester Local Care Organisation) who were members of the Project Management Group (PMG). Thank you to our local Principal Investigators, Louise O'Connor (Lead Nurse for Tissue Viability and Infection Prevention, Manchester University NHS Foundation Trust, who was also a member of the PMG), Joanne Taylor (Tissue Viability Service Lead, Rochdale Care Organisation) and Martin Sylvester (Research Nurse, Mid Yorkshire Hospitals NHS Trust). We also thank our patient representative on the PMG, who has contributed to the work from the outset, including development of the protocol through to interpretation of the findings. The University of Manchester Wounds Research Patient and Public Involvement (PPI) Forum made useful comments on the interview schedule. We thank Francisco Espinoza for setting up the online participant registration form, and the nurse managers at participating Trusts who facilitated recruitment of their staff members as participants. Finally we would like to thank all of the

For peer review only - http://bmjopen.bmj.com/site/about/guidelines.xhtml

staff who so generously gave their time to participate in this research during a very difficult period for NHS services.

Contributors

NC conceived the idea and design for the overall project. RAA, JCD, JG, JFL, and PMW contributed to further development of the study design. CP collected the data. CP, RAA, JG and PMW conducted the data analysis. CP created the original draft of the manuscript. All authors contributed to the interpretation of study findings, critical revision of the manuscript for important intellectual content and approval of the final manuscript.

Competing interests None declared.

Funding

This work was supported by National Institute for Health Research (NIHR) Research for Patient Benefit (RfPB) scheme, grant number PB-PG-0817-20002. NC, JCD, PMW and CP are partially funded by the National Institute for Health and Care Research Applied Research Collaboration Greater Manchester. The views expressed in this publication are those of the author(s) and not necessarily those of the National Institute for Health and Care Research or the Department of Health and Social Care.

Data availability statement

Requests for access to data should be addressed to the corresponding author. Data underlying the study findings will be available on reasonable request. Due to the nature of the ethical approval given for the study it may not be possible to share compete interview transcripts, but additional anonymised illustrative quotations may be available.

REFERENCES

1. NICE. *Varicose veins: diagnosis and management. Clinical guideline.* London: National Institute for Health and Care excellence, 2013.

2. Herber OR, Schnepp W, Rieger MA. A systematic review on the impact of leg ulceration on patients' quality of life. *Health Qual Life Outcomes* 2007;5:44.

3. Green J, Jester R. Health related quality of life and chronic venous leg ulceration: part 1. *Br J Community Nurs* 2009*;*14:S12-S17. doi: 10.12968/bjcn.2009.14.Sup6.45538

For peer review only

For peer review only - http://bmjopen.bmj.com/site/about/guidelines.xhtml

4. Green J, Jester R. Health related quality of life and chronic venous leg ulceration: part 2. Br J Community Nurs 2010;15:S12-S17. doi: 10.12968/bjcn.2010.15.Sup1.46906

5. Cullum N, Buckley H, Dumville J, *et al.* Wounds research for patient benefit: a 5-year programme of research. *Programme Grants for Applied Research 2016;*4:1-304.

6. O'Meara S, Cullum N, Nelson EA, *et al.* Compression for venous leg ulcers. *Cochrane Database Syst Rev* 2012;11:CD000265.

7. SIGN. *Management of chronic venous leg ulcers*: *A national clinical guideline (120):* Scottish Intercollegiate Guidelines Network, 2010.

8. National Wound Care Strategy Programme. *Improving Wound Care.* Accessed 12/01/22 https://www.nationalwoundcarestrategy.net/improving-wound-care/

9. Ashby RL, Gabe R, Ali S, *et al.* VenUS IV (Venous leg Ulcer Study IV) - compression hosiery compared with compression bandaging in the treatment of venous leg ulcers: a randomised controlled trial, mixed-treatment comparison and decision-analytic model. *Health Technol Assess* 2014;18:1-294.

10. Nelson EA, Bell-Syer SEM. Compression for preventing recurrence of venous ulcers. *Cochrane Database Syst Rev* 2012;8:CD002303.

11. Gray TA, Rhodes S, Atkinson RA, *et al.* (2018). Opportunities for better value wound care: a multiservice, cross-sectional survey of complex wounds and their care in a UK community population. *BMJ Open* 2018;8:e019440. doi:10.1136/bmjopen-2018-024859

12. Phillips P, Lumley E, Duncan R, *et al.* A systematic review of qualitative research into people's experiences of living with leg ulcers. *J Adv Nurs* 2018;74:550–563. doi: 10.1111/jan.13465

For peer review only - http://bmjopen.bmj.com/site/about/guidelines.xhtml

13. Weller CD, Buchbinder R, Johnston RV. Interventions for helping people adhere to compression treatments for venous leg ulceration. *Cochrane Database Syst Rev* 2016;3:CD008378.

14. Atkins L, Francis J, Islam R, *et al.* A guide to using the Theoretical Domains Framework of behaviour change to investigate implementation problems. *Implement Sci* 2017;12:77. doi: 10.1186/s13012-017-0605-9

15. Cane J, O'Connor D, Michie S. Validation of the theoretical domains framework for use in behaviour change and implementation research. *Implement Sci* 2012;7:37.

16. Francis JJ, Johnston M, Robertson C, *et al.* What is an adequate sample size? Operationalising data saturation for theory-based interview studies. *Psychol Health* 2010;25:1229-45.

17. Denscombe M. The good research guide for small scale social research projects. Philadelphia: Open University Press 2003:(2nd ed).

18. Bryman A. Social research methods. Oxford United Kingdom: Oxford University Press 2008: (3rd ed).

19. Britten N. Qualitative research. Qualitative interviews in medical research. *BMJ* 1995;311:251-253.

20. Gibbs G. Analyzing qualitative data. London United Kingdom: Sage 2007.

21. Ritchie J, Spencer L, O'Connor W. (2003). Carrying out qualitative analysis. In: Ritchie J, Lewis J, eds. Qualitative research in practice: A guide for social science students and researchers. CA: Sage 2003:220-262.

22. Tobin GA, Begley CM. Methodological rigour within a qualitative framework. *J Adv Nurs* 2004;48:388–396.

23. Weller CD, Richards C, Turnour AM, *et al.* Barriers and enablers to the use of venous leg ulcer clinical practice guidelines in Australian primary care: A qualitative study using the theoretical domains framework. *Int J Nurs Stud* 2019;103.

For peer review only - http://bmjopen.bmj.com/site/about/guidelines.xhtml

24. Franks PJ, Barker J, Collier M, *et al.* Management of patients with venous leg ulcers. Challenges and current best practice. *J Wound Care* 2016;25:s1-s67.

25. Swift A, Punshon G. *District Nursing Today. The view of District Nurse Team Leaders in the UK.* London: The Queen's Nursing Institute, International Community Nursing Observatory, 2019.

26. Nuffield Trust. *The NHS workforce in numbers. Facts on staffing and staff shortages in England.* Accessed 09/11/21www.nuffieldtrust.org.uk/resource/the-nhs-workforce-in-numbers

27. QNI. *Report on District Nurse Education in the United Kingdom 2017 – 2018.* London: The Queen's Nursing Institute, 2019.

28. CLAHRC GM. *ILUMIN: The Leg Ulcer Quality Improvement Programme Summary Report.* Collaboration for Leadership in Applied Health Research and Care Greater Manchester, 2019.

29. NICE. *Overview of management of a person with a venous leg ulcer.* Accessed 30/11/21https://cks.nice.org.uk/topics/leg-ulcer-venous/management/venous-leg-ulcers/#when-to-refer

30. Oates O, Adderley U. Survey of registered nurses' selection of compression systems for the treatment of venous leg ulcers in the UK. *J Tissue Viability* 2019;28:115–119. doi.org/10.1016/j.jtv.2019.02.004

31. Annells M, O'Neill J, Flowers C. Compression bandaging for venous leg ulcers: the essentialness of a willing patient. *J Clin Nurs* 2008;17:350-359. doi: 10.1111/j.1365-2702.2007.01996.x

32. Douglas V. Living with a chronic leg ulcer: an insight into patients' experiences. *J Wound Care* 2001;10:355-360.

33. Van Hecke A, Verhaeghe S, Grypdonck M, *et al.* Processes underlying adherence to leg ulcer treatment: A qualitative field study. *Int J Nurs Stud* 2011;48:145-155.

For peer review only

Barriers and facilitators to compression therapy for people with venous leg ulcers – staff interview schedule

As part of this project we're really interested in understanding the barriers and facilitators to the delivery of compression therapy for people with venous leg ulcers, including factors that may operate at an individual, team and organisation level. We are also interested in your perspectives on how the behaviours of patients themselves might impact positively or negatively on compression use.

Question	Prompts	Rationale/Theoretical Domains Framework (TDF) domains
**Can you tell me a little bit about your current role and your main responsibilities?**	• *How long have you been in this role?*<br>• *What did you do before?*<br>• *How is your team/clinic set up and organised?*	Easy first question, scene setting, supplements demographic information supplied, may reveal something important in relation to role which can be probed later (e.g. 'go to' leg ulcer person).
**From your perspective, is there a 'typical' leg ulcer patient? Could you describe them to me?**	• *Age/sex?*<br>• *Co-morbidities?*<br>• *Lifestyle factors?*	Scene setting.
**Can you tell me what 'gold standard' care and treatment for a patient with a venous leg ulcer looks like?**	• *Vascular assessment.*<br>• *Cleansing and debridement.*<br>• *Dressings and topical treatments.*<br>• *Compression therapy.*<br>• *Systemic therapy.*<br>• *Provision of care.*	Establishing something about interviewees' knowledge/experience – the background against which they practice.<br><br>TDF: knowledge; professional role and identity.
**What guides your choices about venous leg ulcer care?**	• *Knowledge and skills – training, peers, experience, preferences?*<br>• *Local policies?*	TDF: knowledge; skills; professional role and identity; beliefs about capabilities; optimism; beliefs about consequences;

For peer review only - http://bmjopen.bmj.com/site/about/guidelines.xhtml

Question	Prompts	Rationale/Theoretical Domains Framework (TDF) domains
	• *Research evidence - Scottish Intercollegiate Guidelines Network (SIGN) guidelines, Cochrane reviews, reading journals, online searches, specialist peers?*<br>• *Patient and carers' influence – lifestyle, preference, adherence.*	reinforcement; intentions; memory, attention and decision processes; environmental context and resources; social influences; emotion; behavioural regulation.
**What is your experience of offering and delivering compression therapy to patients with venous leg ulcers?**	• *How important do you think compression is?*<br>• *What guides your decision to offer compression? What are your thought processes?*<br>• *How do you explain it to patients and what information do you routinely give them (any patient information/leaflets given)?*<br>• *Do you think patients understand the importance of it?*<br>• *Is there a particular type of compression you like?*<br>• *Do you start on full compression and reduce if patient cannot tolerate/start low and work up to full? Why?*<br>• *How easy or difficult do you find it to deliver compression therapy?*<br>• *What do you think are the necessary skills for effective compression delivery?*<br>• *Have you received relevant training to deliver compression therapy?*<br>• *Are there times when you would choose not to offer or deliver compression (even if it was clinically indicated)?*	TDF: knowledge; skills; professional role and identity; beliefs about capabilities; optimism; beliefs about consequences; reinforcement; intentions; goals; memory, attention and decision processes; environmental context and resources; social influences; emotion; behavioural regulation.
**I'd like to ask you specifically about what you see as the barriers to offering and delivering compression therapy, and what the facilitators are.**<br><br>**Are barriers and facilitators to offering compression therapy different to those**	***Individual level***<br>• *Do you personally find it easy/difficult to offer and deliver compression therapy?*<br>• *How important do you think the ABPI measurement is?*<br>• *What do you do if you are looking after a patient who has not had an ABPI measurement taken?*<br>• *How confident are you in using a Doppler?*	TDF: knowledge; skills; beliefs about capabilities; emotion.

For peer review only - http://bmjopen.bmj.com/site/about/guidelines.xhtml

Question	Prompts	Rationale/Theoretical Domains Framework (TDF) domains
**of delivering compression therapy (may want to address separately if so)?**<br><br>**It may be easier to look at barriers and facilitators in turn. Barriers? Facilitators?**	• *How confident are you in offering and delivering compression therapy?*<br>• *Is there anything that would make you more confident?*<br><br>***Team level***<br>• *Would any other team members influence whether or not you offer or deliver compression therapy (who and how)?*<br>***Organisational level***<br>• *Are there aspects of the community/clinic environment which influence whether or not you are able to offer and deliver compression?*<br>• *Are there any time constraints that influence whether or not you are able to offer and deliver compression?*<br>• *Are there any competing tasks that influence whether or not you are able to offer and deliver compression?*<br>• *Are there any specific issues (e.g. staffing) that influence whether or not you are able to offer and deliver compression?*<br>*If yes to any of the above – how big an issue is this, how does this issue affect your decision making, do you use simpler techniques/interventions so that less experienced staff can then continue using them?*<br>• *What systems are in place to assist with compression use (how easy or difficult is it to deliver compression)?*<br>• *What availability is there of Dopplers; staff training; patient information leaflets?*	TDF: professional role and identity; reinforcement; social influences.<br>TDF: professional role and identity; reinforcement; intentions; environmental context and resources; social influences; behavioural regulation.
**How might the views of your patients affect how you offer and deliver compression?**	• *Does compression therapy influence your relationships with patients? (does it affect communication?)*<br>• *How do you respond when a patient refuses to wear compression/is non-adherent?*<br>• *Do your colleagues generally agree with you about this approach to patient non-adherence?*	TDF: beliefs about consequences; goals; environmental context and resources; social influences; emotion.

For peer review only

Question	Prompts	Rationale/Theoretical Domains Framework (TDF) domains
	• *Do you find that you change from full compression to reduced compression?*<br>• *What are your views about reduced compression?*<br>• *Do you find patients adhere to reduced compression?*<br>• *Does not being able to deliver compression cause you to be worried or concerned?*	
**Have you any views on why patients do or do not adhere to their compression therapy?**	• *Patient discomfort?*<br>• *Patient lifestyle?*<br>• *Patient beliefs about illness and treatment?*<br>• *Influence of partner/family/friends?*<br>• *Do your colleagues generally agree with you about patient non-adherence?*	TDF: beliefs about consequences; goals; environmental context and resources; social influences; emotion.
**What do you perceive to be the consequences of offering and delivering compression therapy?**	• *Does it influence the outcomes?*<br>• *Does it have an impact on your clinical workload and patient routines?*<br>• *What happens if you are not able to deliver compression as you would like to? (to patients, to colleagues, to yourself? Short and long-term consequences?)*	TDF: beliefs about consequences; optimism; intentions; goals; social influences; emotion.
**What would help you to overcome any difficulties/challenges that you perceive in offering and delivering compression therapy?**	• Skills training in using a Doppler machine?<br>• Other skills training?<br>• The number of Dopplers available?<br>• Other equipment?<br>• *What would you personally have to do to increase the number of patients wearing compression? (in the ideal world).*<br>• *If you wanted to implement changes in your own practice to increase the number of patients wearing compression, what would be some of the ways to do this?*	TDF: knowledge; skills; beliefs about capabilities; optimism; reinforcement; memory, attention and decision processes; environmental context and resources; behavioural regulation.

For peer review only - http://bmjopen.bmj.com/site/about/guidelines.xhtml

Question	Prompts	Rationale/Theoretical Domains Framework (TDF) domains
**Is there anything else about your experience of offering and delivering compression therapy that I haven't asked you about that you think it is important to say?**		Tapping into knowledge/experience of individual.
**Is there anybody else that you can think of (individual/groups) who you think it might be useful for me to talk to as part of this study?**		Snowballing/tapping into knowledge/experience of individual.

For peer review only

COREQ (Consolidated criteria for Reporting Qualitative research) Checklist

Topic	Item No.	Guide Questions/Description	Reported on Page No.
**Domain 1: Research team and reflexivity**			
Personal characteristics			
Interviewer/facilitator	1	Which author/s conducted the interview or focus group?	7 (resubmitted manuscript)
Credentials	2	What were the researcher's credentials? E.g. PhD, MD	Supplementary material below
Occupation	3	What was their occupation at the time of the study?	Supplementary material below
Gender	4	Was the researcher male or female?	Supplementary material below
Experience and training	5	What experience or training did the researcher have?	Supplementary material below
Relationship with participants			
Relationship established	6	Was a relationship established prior to study commencement?	Supplementary material below
Participant knowledge of interviewer	7	What did the participants know about the researcher? E.g. personal goals, reasons for doing the research.	Supplementary material below
Interviewer characteristics	8	What characteristics were reported about the interviewer/facilitator? E.g. bias, assumptions, reasons and interests in the research topic.	Supplementary material below
**Domain 2: Study design**			
Theoretical framework			
Methodological orientation and theory	9	What methodological orientation was stated to underpin the study? E.g. grounded theory, discourse analysis, ethnography, phenomenology, content analysis.	7 (resubmitted manuscript)
Participant selection			
Sampling	10	How were participants selected? E.g. purposive, convenience, consecutive, snowball.	7 (resubmitted manuscript)
Method of approach	11	How were participants approached? E.g. face-to-face, telephone, mail, email.	7 (resubmitted manuscript)
Sample size	12	How many participants were in the study?	8 (resubmitted manuscript)
Non-participation	13	How many people refused to participate or dropped out? Reasons?	7 (resubmitted manuscript) and supplementary material below
Setting			
Setting of data collection	14	Where was the data collected? E.g. home, clinic, workplace.	7 (resubmitted manuscript)
Presence of non-participants	15	Was anyone else present besides the participants and researchers?	N/A

For peer review only - http://bmjopen.bmj.com/site/about/guidelines.xhtml

Topic	Item No.	Guide Questions/Description	Reported on Page No.
Description of sample	16	What are the important characteristics of the sample? E.g. demographic data, date.	7 and 8-10 (resubmitted document)
Data collection			
Interview guide	17	Were questions, prompts, guides provided by the authors? Was it pilot tested?	7 (resubmitted document) and Supplementary File 1
Repeat interviews	18	Were repeat interviews carried out? If yes, how many?	N/A
Audio/visual recording	19	Did the research use audio or visual recording to collect the data?	7 (resubmitted document)
Field notes	20	Were field notes made during and/or after the interview or focus group?	Supplementary material below
Duration	21	What was the duration of the interviews or focus group?	8 (resubmitted document)
Data saturation	22	Was data saturation discussed?	7 (resubmitted document)
Transcripts returned	23	Were transcripts returned to participants for comment and/or correction?	Supplementary material below
**Domain 3: analysis and findings**			
Data analysis			
Number of data coders	24	How many data coders coded the data?	7-8 (resubmitted document)
Description of the coding tree	25	Did the authors provide a description of the coding tree?	11 (resubmitted document)
Derivation of themes	26	Were themes identified in advance or derived from the data?	7 (resubmitted manuscript)
Software	27	What software, if applicable, was used to manage the data?	7 (resubmitted manuscript)
Participant checking	28	Did participants provide feedback on the findings?	8 (resubmitted document)
Reporting			
Quotations presented	29	Were participant quotations presented to illustrate the themes/findings? Was each quotation identified? E.g. participant number.	11 (resubmitted manuscript) and throughout results
Data and findings consistent	30	Was there consistency between the data presented and the findings?	Throughout results
Clarity of major themes	31	Were major themes clearly presented in the findings?	Throughout results
Clarity of minor themes	32	Is there a description of diverse cases or discussion of minor themes?	Throughout results

For peer review only - http://bmjopen.bmj.com/site/about/guidelines.xhtml

Developed from: Tong A, Sainsbury P, Craig J. Consolidated criteria for reporting qualitative research (COREQ): a 32 item checklist for interviews and focus groups. *International Journal for Quality in Health Care.* 2007. Volume 19, Number 6: pp.349-357.

Supplementary material
Below is presented additional detail about issues that it was not possible to present in the main paper, arranged under the COREQ headings.

Research team and reflexivity
Please see page 7 (resubmitted paper), with supplementary material below.

Personal characteristics
Interviews were conducted by one researcher (CP). She was female, held a PhD, and was an experienced interviewer who had received training in qualitative research methods over a number of years, including during the conduct of her PhD. At the time of the study the researcher was employed as a University Research Fellow.

Relationship with participants
There was no relationship between research participants and the researcher prior to the commencement of the study. Potential participants had been provided with details of the study by their manager and if they were interested in learning more had completed an on-line form. They were then contacted via email by the researcher. The researcher's role as a University researcher was explained to individual potential interviewees. It was ensured that researcher contact details were clearly available in case potential participants had further questions.

Study design
Please see main pages 7-8 (resubmitted paper), with supplementary material below.

Method of approach
We liaised with the nurse managers to ensure that they used a standardised text to approach their staff, this text was approved by the NHS Ethics Committee who gave ethical approval for the research. The nurse managers sent out the invite only once and did not follow it up, and would not know who did or did not respond to the invitation.

Participant selection
Eighteen potential participants registered their interest in participating in the study; three of these people did not respond to interview invitations and so were not interviewed.

Data collection
We developed a semi-structured interview schedule (Supplementary File 1) with reference to the research aim, the literature, and the Theoretical Domains Framework. This schedule was 'sense checked' by practitioner members of the study team. The interview schedule was kept under review as the interviews progressed to ensure that we were generating relevant data. Interview participants were offered sight of transcripts, although nobody availed themselves of this. Field notes were used to provide context.

Analysis and findings
Please see pages 7-11 (resubmitted paper) and throughout results.

For peer review only - http://bmjopen.bmj.com/site/about/guidelines.xhtml

BMJ Open

What promotes or prevents greater use of appropriate compression in people with venous leg ulcers? A qualitative interview study with nurses in the north of England using the Theoretical Domains Framework

Journal:	*BMJ Open*
Manuscript ID	bmjopen-2022-061834.R2
Article Type:	Original research
Date Submitted by the Author:	06-Jul-2022
Complete List of Authors:	Perry, Catherine ; The University of Manchester, Division of Population Health, Health Services Research and Primary Care, School of Health Sciences<br>Atkinson, Ross; The University of Manchester, Division of Nursing, Midwifery and Social Work, School of Health Sciences, Faculty of Biology, Medicine and Health<br>Griffiths, Jane; The University of Manchester, Division of Nursing, Midwifery and Social Work, School of Health Sciences<br>Wilson, Paul; University of Manchester, Division of Population Health, Health Services Research and Primary Care, School of Health Sciences<br>Lavallée, Jacqueline; The University of Manchester, Division of Medical Education, School of Medical Sciences<br>Mullings, Julie; Manchester University NHS Foundation Trust<br>Cullum, Nicky; The University of Manchester, School of Health Sciences; Manchester University NHS Foundation Trust, MAHSC<br>Dumville, Jo C.; The University of Manchester, Division of Nursing, Midwifery and Social Work, School of Health Sciences
**Primary Subject Heading**:	Nursing
Secondary Subject Heading:	Nursing, Qualitative research
Keywords:	Vascular medicine < INTERNAL MEDICINE, PRIMARY CARE, QUALITATIVE RESEARCH, WOUND MANAGEMENT

SCHOLARONE™
Manuscripts

I, the Submitting Author has the right to grant and does grant on behalf of all authors of the Work (as defined in the below author licence), an exclusive licence and/or a non-exclusive licence for contributions from authors who are: i) UK Crown employees; ii) where BMJ has agreed a CC-BY licence shall apply, and/or iii) in accordance with the terms applicable for US Federal Government officers or employees acting as part of their official duties; on a worldwide, perpetual, irrevocable, royalty-free basis to BMJ Publishing Group Ltd ("BMJ") its licensees and where the relevant Journal is co-owned by BMJ to the co-owners of the Journal, to publish the Work in this journal and any other BMJ products and to exploit all rights, as set out in our licence.

The Submitting Author accepts and understands that any supply made under these terms is made by BMJ to the Submitting Author unless you are acting as an employee on behalf of your employer or a postgraduate student of an affiliated institution which is paying any applicable article publishing charge ("APC") for Open Access articles. Where the Submitting Author wishes to make the Work available on an Open Access basis (and intends to pay the relevant APC), the terms of reuse of such Open Access shall be governed by a Creative Commons licence – details of these licences and which Creative Commons licence will apply to this Work are set out in our licence referred to above.

Other than as permitted in any relevant BMJ Author's Self Archiving Policies, I confirm this Work has not been accepted for publication elsewhere, is not being considered for publication elsewhere and does not duplicate material already published. I confirm all authors consent to publication of this Work and authorise the granting of this licence.

For peer review only

What promotes or prevents greater use of appropriate compression in people with venous leg ulcers? A qualitative interview study with nurses in the north of England using the Theoretical Domains Framework

Authors

Catherine Perry, Division of Population Health, Health Services Research and Primary Care, School of Health Sciences, University of Manchester, UK.

Ross A Atkinson, Division of Nursing, Midwifery and Social Work, School of Health Sciences, University of Manchester, UK.

Jane Griffiths, Division of Nursing, Midwifery and Social Work, School of Health Sciences, University of Manchester, UK.

Paul M Wilson, Division of Population Health, Health Services Research and Primary Care, School of Health Sciences, University of Manchester, UK.

Jacqueline F Lavallée, Division of Medical Education, School of Medical Sciences, University of Manchester, UK.

Julie Mullings, Manchester University NHS Foundation Trust, Manchester, UK.

Nicky Cullum, Division of Nursing, Midwifery and Social Work, School of Health Sciences, University of Manchester, and MAHSC, Manchester University NHS Foundation Trust, Manchester, UK.

Jo C Dumville, Division of Nursing, Midwifery and Social Work, School of Health Sciences, University of Manchester, UK.

Correspondence to:

Catherine Perry, Division of Population Health, Health Services Research and Primary Care, School of Health Sciences, University of Manchester, UK

Catherine.Perry@manchester.ac.uk

For peer review only - http://bmjopen.bmj.com/site/about/guidelines.xhtml

ABSTRACT

Objectives

To investigate factors that promote and prevent the use of compression therapy in people with venous leg ulcers.

Design

Qualitative interview study with nurses using the Theoretical Domains Framework (TDF).

Setting

Three NHS Trusts in England.

Participants

Purposive sample of 15 nurses delivering wound care.

Results

Nurses described factors which made provision of compression therapy challenging. Organisational barriers (TDF domains environmental context and resources/knowledge, skills/behavioural regulation) included heavy/increasing caseloads; lack of knowledge/skills and the provision of training; and prescribing issues (variations in bandaging systems/whether nurses could prescribe). Absence of specialist leg ulcer services to refer patients into was perceived as a barrier to providing optimal care by some community-based nurses. Compression use was perceived to be facilitated by clinics for timely initial assessment; continuity of staff and good liaison between vascular/leg ulcer clinics and community teams; clear local policies and care pathways; and opportunities for training such as 'shadowing' in vascular/leg ulcer clinics. Patient engagement barriers (TDF domains goals/beliefs about consequences) focussed on getting patients 'on board' with compression, and supporting them in using it. Clear explanations were seen as key in promoting compression use.

Conclusions

Rising workload pressures present significant challenges to enhancing leg ulcer services. There may be opportunities to develop facilitated approaches to enable community nursing teams to make changes to practice, enhancing quality of patient care. The majority of venous leg ulcers could be managed in the community without referral to specialist community services if issues relating to workloads/skills/training are addressed. Barriers to promoting compression use could also be targeted, for example through the development of clear patient information leaflets. Whilst patient engagement barriers may be easier/quicker to address than organisational barriers, unless organisational barriers are addressed it seems unlikely that all people who would benefit from compression therapy will receive it.

For peer review only

For peer review only - http://bmjopen.bmj.com/site/about/guidelines.xhtml

Strengths and limitations of this study

- Using qualitative interviews with nurses delivering wound care enabled an in-depth exploration of what promotes or prevents the use of compression therapy in people with venous leg ulcers, from the perspective of staff.
- Initial inductive analysis of interview data ensured that all data were accounted for, and the use of the Theoretical Domains Framework provided a theoretical structure for understanding factors that promoted or prevented compression us.
- Participating nurses were from three Trusts (provider organisations) in the North of England; staff in other geographical regions may have other views.
- All interviews were conducted over the telephone due to the COVID-19 pandemic, so those less happy with using the telephone may not have participated.

INTRODUCTION

Background

Venous leg ulcers (VLUs) are common, open wounds on the lower leg which take more than two weeks to heal.[1] Problems with the veins in the leg (such as damage to the valves, or blockages), or impaired action of the calf muscles, prevent optimal blood flow to the heart. This causes venous stasis, increasing the pressure in the veins and triggering a complex change in tissue physiology that can result in open wounds which heal slowly, if at all. Leg ulcers are more prevalent in older people. They can be painful, impair mobility and sleep, restrict work and leisure activities and reduce psychological well-being, impacting greatly on health-related quality of life.[2-4] Complete wound healing has been identified as the main outcome desired by people with VLUs.[5]

In the UK, VLU care is commonly delivered in the community, in patients' homes or clinics, by nurses or other health professionals. Whilst a wide range of largely nurse-delivered community-focused interventions are available to treat VLUs (bandages, hosiery, dressings and topical agents among others), only compression therapy is supported by a convincing level of evidence[6] and recommended by clinical guidelines.[7 8] Compression therapy, in the form of bandages or stockings, is cost-effective and has become the first-line, evidence-based treatment for VLUs.[6 9] In addition, compression hosiery is current standard practice for prevention.[10]

Compression works when used as intended, but delivery and adherence can be problematic. For example, previous research has highlighted unwarranted variation in clinical practices

For peer review only - http://bmjopen.bmj.com/site/about/guidelines.xhtml

across wound care services in the north of England.[11] This included significant underuse of compression therapy where clinically appropriate. Understanding how and why sub-optimal delivery of evidence-based practices happens is an essential prerequisite to developing strategies to enhance the quality of care delivered. Whilst there is recognition that patient factors may play a role, including discomfort, patients' understanding of the causes of ulceration, and their beliefs about compression in terms of healing and symptom control,[12, 13] less is known about the influences on professional behaviours. The aim of this study was to explore, from the perspective of nurses delivering wound care, the factors that promote or prevent the use of compression therapy for people with VLUs.

Theoretical underpinning

Understanding existing professional practices with a view to promoting positive changes in individual and collective behaviour requires an understanding of the influences on behaviours in the context in which they occur.[14] The Theoretical Domains Framework (TDF) is a comprehensive, theory-informed approach for identifying determinants of professional behaviour (cognitive, affective, social and environmental).[11 12] Organised across 14 conceptual domains (see Table 1), the TDF has been applied across several healthcare settings to explore professional practices and behaviours.[14 15] We used the TDF to investigate the factors that promote and prevent the use of compression therapy as a treatment for VLUs; thus identifying potentially modifiable determinants of implementation and providing a theoretical foundation to link these to targets for behaviour change.

For peer review only

Table 1. The Theoretical Domains Framework: domains and definitions

Domain	Definition
Knowledge	An awareness of the existence of something
Skills	An ability or proficiency acquired through practice
Social/professional role and identity	A coherent set of behaviours and displayed personal qualities of an individual in a social or work setting
Beliefs about capabilities	Acceptance of the truth, reality, or validity about an ability, talent or facility that a person can put to constructive use
Optimism	The confidence that things will happen for the best or that desired goals will be attained
Beliefs about consequences	Acceptance of the truth, reality or validity about the outcomes of a behaviour in a given situation
Reinforcement	Increasing the probability of a response by arranging a dependent relationship or contingency between the response and a given stimulus
Intentions	A conscious decision to perform a behaviour or a resolve to act in a certain way
Goals	Mental representations of outcomes and states that an individual wants to achieve
Memory, attention and decision processes	The ability to retain information, focus selectively on aspects of the environment and choose between two or more alternatives
Environmental context and resources	Any circumstance of a person's situation or environment that discourages or encourages the development of skills and abilities, independence, social competence and adaptive behaviour
Social influences	Those interpersonal processes that can cause individuals to change their thoughts, feelings or behaviours
Emotion	A complex reaction pattern, involving experiential, behavioural, and psychological elements, by which the individual attempts to deal with a personally significant matter or event
Behavioural regulation	Anything aimed at managing or changing objectively observed or measured actions

For peer review only - http://bmjopen.bmj.com/site/about/guidelines.xhtml

METHODS

Sample

We purposively sampled participants from three NHS Trusts in the North of England (Trust 1 comprised 12 community nursing teams, one community leg ulcer clinic and one secondary care specialist clinic; Trust 2 six community nursing teams; Trust 3 11 community nursing teams). All were qualified nurses providing leg ulcer care within the community, including community nurses and nurses who worked in hospital outpatient clinics.

Participant recruitment and data generation

Recruitment and data generation took place between February 2020 and January 2021. Potential participants were contacted by their manager, who provided them with a brief description of the study and a link to an online form to register their interest in participating. Those completing the online form were contacted via email, including a Participant Information Sheet, by a researcher. If they were happy to participate a date and time were arranged for interview. Three potential participants who completed the online form did not go on to interview. Sampling was continued until it was judged that data saturation had been reached.[16]

We had planned to conduct face-to-face interviews, however, because of COVID-19 restrictions, all interviews were conducted over the telephone. Verbal consent was taken and digitally audio-recorded.

We undertook semi-structured interviews as they offer a good way to generate data on individuals' experiences.[17] We developed a semi-structured interview schedule (Supplementary File 1) with reference to the research aim, the literature, and the TDF. Interview questions defined the area to be explored[18] but allowed the interviewer or interviewee to diverge in order to follow up particular areas in more detail.[19] With the permission of participants, interviews were digitally audio-recorded and transcribed.

Data analysis

We uploaded interview transcripts onto NVivo software to aid data management.[20] Initially, an inductive thematic analysis was undertaken to create a framework for the data.[21] This was followed by a deductive analysis in which the data from the framework were mapped on to the TDF domains

The initial inductive analysis was carried out by the lead qualitative researcher who had conducted all of the interviews (CP) and to ensure dependability[22] the developing analytical

For peer review only - http://bmjopen.bmj.com/site/about/guidelines.xhtml

framework was shared and discussed with the research team. Similarly, the deductive analysis mapping specific barriers and enablers within each TDF domain was carried out by one author (CP) in regular discussion with the research team, with additional sense-making and insight provided by the Study Steering Group. To ensure credibility[22] a near final version of the analysis was presented to a group of 14 nurses experienced in wound care from across England during an online workshop arranged by the National Wound Care Strategy Programme.[8] They were asked about their views on the barriers to compression therapies identified in the study and how best practice may be enabled, and agreed that the findings (and our interpretation of them) reflected their own experiences and/or made sense to them.

Patient and public involvement

A patient representative sits on the Study Steering Group and has been an active member of the team, contributing to the work from the writing of the study protocol through to discussion of emerging findings and their interpretation. The study was also presented to the University of Manchester Wounds Research Patient and Public Involvement (PPI) Forum. In particular members of the forum were consulted about interview schedule questions covering issues pertinent to them, for example questions regarding the importance of the staff-patient relationship.

RESULTS

Participant characteristics

We interviewed 15 nurses who delivered wound care, with interviews between 25 and 69 minutes in length. Interviewees were all female and comprised nine community nurses or managers, three vascular nurse specialists and three leg ulcer nurses, with a range of experience represented (Table 2).

For peer review only

Table 2. Participant characteristics

Participant ID (cl clinic-based role, co community-based role)	Job title (self-reported)	Length of service as qualified nurse (years)	Time in current role (years)	Highest educational qualification	Training
001cl	Sister, Community Leg Ulcer Service	31-40	11-20	Diploma	N18 (compression therapy)<br>V150 (prescribing)
002co	Community Specialist Practitioner Student	6-10	6-10	Currently completing BSc in District Nursing	'In house' leg ulcer training
003cl	Vascular Leg Ulcer Nurse	21-30	0-5	BSc	Tissue viability and leg ulcer management modules (accredited)<br>Ongoing wound management, leg ulcer updates, courses, conferences.
004cl	Vascular Nurse Specialist	11-20	6-10	MSc	Leg ulcer management (University, stand-alone module)<br>Wound debridement (University, stand-alone module)<br>Attendance at Wounds UK Conferences
005co	Community Staff Nurse	11-20	6-10	Diploma in Nursing with advanced standing	Leg ulcer management Level 6<br>V150 (prescribing)<br>Online wound care training sessions
006co	Community Staff Nurse	21-30	11-20	BSc (hons) Nursing Studies	N18 leg ulcer course<br>Leg ulcer conferences including Lindsay Leg Club<br>Annual ‘in-house’ wound care training
007cl	Clinical Nurse Specialist (vascular)	31-40	11-20	Diploma in Nursing	Leg Ulcer management<br>Mentorship (supporting learning in practice) Community Practitioner

For peer review only - http://bmjopen.bmj.com/site/about/guidelines.xhtml

					Nurse Prescribing Vascular Disease Management (University)
008co	District Nursing Sister	6-10	6-10	Specialist Practitioner District Nursing	Leg Ulcer Management (University) V150 Community Prescriber.
009co	Leg Ulcer Nurse	21-30	0-5	RGN	Leg Ulcer Management (online, University)
010cl	Leg Ulcer Nurse	11-20	0-5	Diploma in Nursing	Leg Ulcer management Masters module (online)<br>Wound UK courses<br>Lindsay leg Club course
011co	Practice Educator for Community Vascular Services	11-20	0-5	Advanced Diploma in adult Nursing	Leg Ulcer management Masters module (online)<br>Nurse Prescriber for Community Formulary<br>Trust 2 day wound care course,<br>Regular TVN and VNS wound care updates
012co	Senior Community Nurse	0-5	0-5	BA Nursing	Leg Ulcer Course (1 day) run by Urgo<br>'In house' courses<br>Nurse Prescriber
013co	Community Nurse	0-5	0-5	BSc (hons) Nursing	Trust training - wound care and compression
014co	Neighbourhood Lead	11-20	11-20	Masters (Research)	Numerous training courses over years
015co	Community Nurse	6-10	6-10	RGN	Leg ulcer module (10 days)<br>Drug Company study days

BA, Bachelor of Arts; BSc, Bachelor of Science; MSc, Master of Science; RGN, Registered General Nurse; TVN, Tissue Viability Nurse; VNS, Vascular Nurse Specialist.

For peer review only - http://bmjopen.bmj.com/site/about/guidelines.xhtml

Relevant domains and key themes

We coded data to seven TDF domains: environmental context and resources, goals, knowledge, skills, beliefs about consequences, behavioural regulation and intentions. The domains of knowledge and skills are presented together as they were linked in the data by the issue of training. We did not have any data that related to the other TDF domains. We did not expect to have data in all of the domains due to the comprehensive nature of the framework. Domains and their associated themes are displayed in Table 3. The findings are presented in relation to the TDF domains, with the domains to which most data were coded presented first. Quotations from participants are used to illustrate points made, and are identified with a participant ID and indication of whether they are primarily community-based (co) or clinic-based (cl).

Table 3. Domains and associated themes

Domain	Theme
Environmental context and resources	Lack of commissioned leg ulcer services<br>Delays in initial leg and wound assessment<br>Lack of continuity of care<br>'Joined up' services and multidisciplinary teams<br>Prescribing issues<br>Application of compression as physically hard work
Goals	Getting patients 'on board'<br>Explaining timeframes around healing
Knowledge and skills	Gaps in knowledge and skills<br>Training
Beliefs about consequences	Encouraging compression use<br>Response to non-adherence
Behavioural regulation	Variations in bandaging systems used
Intentions	Local policies and leg ulcer pathways<br>Compression as treatment of choice

Environmental context and resources

Lack of commissioned leg ulcer services

In some areas the lack of specifically commissioned leg ulcer services was mentioned as a barrier to the provision of appropriate compression therapy; in these areas leg ulcer care was provided as part of general community nursing services: *'It's just wrapped up in district*

For peer review only - http://bmjopen.bmj.com/site/about/guidelines.xhtml

nursing.' (014co). Community nurses discussed the challenge of offering optimal care, either because of perceived lack of skills in compression therapy or because of other responsibilities, resulting in apparently eligible people not receiving compression: *'You're not able to follow up….and like offer the treatment as they should anyway because of all the other things you have to worry about.'* (002co). Non-housebound patients were not eligible for home visits from community nursing services; in some areas they could attend community nurse-led clinics but in others they received leg ulcer care from practice nurses at their GP surgery, and these nurses were perceived as less likely to be trained in compression therapy: *'Sometimes the practice nurses are not competent to do the compression bandaging.'* (004cl). These issues could affect the type of compression a patient was offered.

In areas with commissioned leg ulcer services and leg ulcer clinics, the clinics were highly thought of and seen as facilitating good quality care, incorporating appropriately trained staff and continuity:

> *'The leg ulcer clinics in [name of areas] are fabulous. Fabulous for patients, great for continuity, but also, great for anybody who has joined the district nursing team to make sure we've got that consistency across the* [area]*.'* (014co).

Questions were, however, raised about the accessibility of some clinics, and it was suggested that patients may have difficulty getting to clinic locations on multiple/frequent occasions.

Delays in initial leg and wound assessment

The importance of fast, timely, initial assessment of someone with a suspected VLU was a universal theme: *'Getting the treatment in place quickly, because otherwise it can become….like the chronicity increases and the chance of healing decreases.'* (004cl). However, delays in initial assessment were often described. This could be because community nurses did not recognise the need for specialised assessment of a wound: *'Nobody's thought about doing a Doppler. And that might be because they're not experienced, so hadn't thought about it.'* (006co); or that they lacked the skills to carry it out: *'There's only a select few of us within the team who can complete the Doppler assessment…..there's just not enough of us.'* (005co); (the Doppler assessment measures ankle-brachial pressure index (ABPI) and is an important component of initial assessment prior to compression use). Delays in access to Doppler were also ascribed to the general pressure of work in the community:

For peer review only - http://bmjopen.bmj.com/site/about/guidelines.xhtml

> *'So, pressure of work most definitely is a barrier……you could go in, see the patient…..you know put the dressing on. You are in and out of that house in 15 minutes, aren't you? You can also send your unregistered, or registered but non-nurse members of the team and so, even though our leg ulcer pathway, if you like, says, the gold standard, after two weeks you should be doing a Doppler, pressure of work, if you've got loads and loads of visits and not many staff and you've got low grades of staff, well then, that can slip can't it?'* (014co).

Even in areas with dedicated leg ulcer clinics delays in getting an initial assessment were described:

> *'There needs to be more of them* [leg ulcer clinics]*, because patients are still waiting too long for assessment. So we see lots of patients in vascular* [clinic] *that have waited three, four months. So they've been seeing the district nurses, waiting three or four months to go and see the leg ulcer clinic, and in that time their leg's getting worse and worse and worse, and in the end, the GP's referred them to Vascular. So they've ended up in secondary care, because the waiting lists in primary care are too long.'* (003cl).

Two interviewees highlighted the value of specific assessment clinics which had been established to enable timely assessment and one spoke about a new role that she had undertaken providing community vascular support, which she said could lead to more timely assessments. More broadly, interviewees spoke about promoting awareness of leg care. One talked about *'a Legs Matter week….what they tend to do is promote healthy legs and promote compression and promote good skincare*' (006co) and another suggested *'it's not just patients, just nurses in general need [awareness raising]'* (001cl).

Lack of continuity of care

A lack of continuity of nursing care, with different staff attending patients, was perceived as a barrier to effective compression therapy. It was more a problem for community teams than in leg ulcer clinics, and could result in staff not having an overview of a patient's care, knowledge of their disease or treatment history, or reasoning behind clinical decisions. A nurse explained:

> *'The teams are so large, what will happen is, somebody will start the reduced compression. And then obviously, a different person goes in next time, and they will think they're in reduced compression, because they've got a problem with their arterial line. And no one will ever increase that compression, because they assume, because they're in reduced compression, that they've got some, you know…'* (003cl).

This situation could be exacerbated by the lack of availability of electronic patient records in the community. A vascular clinic nurse commented:

For peer review only - http://bmjopen.bmj.com/site/about/guidelines.xhtml

> *'So that's another thing that adds to it. When everyone's on different [patient record systems]. "We can't access the [electronic] hospital records". I don't think the district nurses have got access. We have but they don't.'* (010cl).

Lack of continuity in staffing was perceived as leading to a situation where *'somebody else thinks somebody else's, you know, it's somebody else's responsibility'* (006co). One interviewee described a change in working practices, 'zonal working' where a smaller group of nurses covered a defined geographical area, as increasing the likelihood of staff continuity. In addition, patients having contact details for the nursing team, for example through a telephone help-line, was thought to enhance continuity of care and communication between staff and patients.

'Joined up' services and multidisciplinary teams

A lack of communication between different services was identified as a barrier to optimal care. A patient with a VLU could be seen by hospital vascular services, leg ulcer clinics and district nursing services, as well as have contact with primary and secondary care services for issues other than their VLU. It was reported that these services did not necessarily communicate well with each other, resulting in unnecessary duplication (e.g., where a patient gets referred to *both* a leg ulcer clinic and a vascular clinic) or unnecessary referral (e.g., where a patient had undergone investigations whilst a hospital inpatient but the results of these investigations were not communicated to the other services). Patients not receiving their compression therapy whilst hospital inpatients was frequently mentioned:

> *'Another barrier to our work is that we can be working really hard with a patient, they become unwell, they're admitted to hospital for something completely different. And the first thing they do is take the compression bandages off their legs and just stick a plaster on. Because nobody within hospital can do that treatment either.'* (001cl).

Conversely, good liaison between vascular or leg ulcer clinics and community nursing teams was described as a facilitator of good care. Often a patient would be seen in a vascular or leg ulcer clinic, a treatment plan drawn up and they would be returned to community teams to continue the treatment. The majority of both clinic-based nurses and community-based nurses reported good communication and liaison between the two: *'Any of the patients now that come to see me leave this clinic with a concise treatment plan…..I've got good established links with community'* (003cl); *'We have the numbers of all the vascular specialist nurses, so if we feel like we just need to ask a little bit of advice… they'll get back to us straight away'* (013co).

For peer review only - http://bmjopen.bmj.com/site/about/guidelines.xhtml

Multidisciplinary teams, including nurses, physiotherapists, podiatrists and social workers working well together were described as facilitating high quality VLU care and compression therapy. The importance of working with the care system was also highlighted in terms of care workers being able to support patients with their compression therapy. One nurse also suggested working with care workers on training:

> *'The care providers, because they're often quite low-paid, so they have minimal training and that's why we're, our vision is joint training. Because, if they could come on our leg ulcer morning or updates, so that they understand the importance of the compression.'* (014co).

Prescribing issues

Compression therapy is a prescribed treatment. Nurses who had been trained as independent prescribers were able to prescribe compression, others had to ask a patient's GP to prescribe the treatment. This could cause delays and could result in patients not having prescribed treatment in time for their next nurse appointment:

> *'[Patient takes] your form to the GP. It has to sit at the GP's for 48 hours. They then do a prescription. The patient has to pick the prescription up, take it to the chemist. You might wait another 48 hours to pick your prescription up.'* (001cl).

Most clinic nurses and some community nurses had stocks of compression which they could use if necessary (patients were often started off in their compression from these stocks), but this was not always the case.

Application of compression as physically hard work

Four interviewees reported that application of compression therapy, in some circumstances, can be physically hard work, particularly where the application takes place in patients' homes without any aids including treatment couches at the correct height. One interviewee commented: *'for district nurses it will often be on the floor on their hands and knees….so it is physically a little bit tricky'* (004cl) and another: *'we've had a couple of staff that had got either bad backs or bad knees. So that takes them out of being able to do it for a couple of weeks'* (012co). Suggested facilitators were working in pairs, and spreading out compression visits over several days in order to avoid having too many on one day, but these solutions often depended on adequate staffing.

Goals

Getting patients 'on board'

For peer review only - http://bmjopen.bmj.com/site/about/guidelines.xhtml

All Interviewees described the importance of getting patients 'on board' with their treatment, that is, in agreement with and involved with it, from the beginning: *'I think if they're not on board from the beginning, you're fighting a losing battle.'* (005co). This could be difficult: *'Getting them on board to comply with the compression, and the correct compression. I think basically, that's the biggest one that we have a problem with.'* (007cl). Explanations were seen as key to engaging patients: nurses were clear that if patients understood that their VLU was a symptom of an underlying long-term condition, and understood the mechanism of compression therapy, they were more likely to adhere to treatment.

In relation to explaining aetiology and compression therapy, interviewees talked about the availability and utility of written information to use with patients. A minority did not use written information at all and some thought that patients did not read information leaflets anyway. However, the majority of interviewees thought that written information could be useful:

> *'So it just gives them something to read at their own leisure, and then next time you go, they've got a bit more of an understanding of what you're talking about as well. So I find the leaflets do work quite well.'* (008co).

Some interviewees had access to 'Trust leaflets' and product company leaflets were also mentioned, although worries were expressed about bias, which may be indicative of variation in the quality of this information. In order to be useful all interviewees thought that leaflets needed to have very simple, clear explanations, some said that pictures were helpful, and one person suggested alternative information formats such as short films.

In terms of getting patients 'on board', the majority of interviewees also spoke of giving patients options or choices, which might make them more likely to adhere to their compression therapy. One nurse explained:

> *'Like this is how I would feel you'd do best, however, these are your other options. And I'd rather give options at this point and formulate a plan, because you tend to get a little bit more concordance than saying well, this is what you are going to do.'* (001cl).

There were a number of situations in which it could be possible to offer options, for example less obtrusive compression or being flexible with clinic appointments. All nurses agreed that it was important to build a relationship with their patient in which they could discuss treatment issues and work together towards the best outcome for the patient: '*A lot of it is getting them on board, building that relationship up with your patients…….and it's a journey together really.'* (007cl); *'It's teamwork, it's not a one-way system.'* (002co).

Explaining timeframes around healing

For peer review only - http://bmjopen.bmj.com/site/about/guidelines.xhtml

The difficulty in estimating how long a VLU may take to heal and explaining this to a patient could be a barrier to setting goals and the successful use of compression therapy. All interviewees said that patients always asked about how long healing would take: *'I think that's one of the first things they'll ask you.'* (001cl). Nurses suggested that giving a specific time frame should be avoided: *'The worst thing you could do is give the time. Because, then they're going to sit and wait and say right it's been this long, and it's not healed.'* (008co). A number of things that interviewees thought helped in explaining timeframes included being positive; talking about healing in small steps; describing what patients can do to speed up healing and issues that may slow healing; and being honest about ulcers that are likely to take a long time to heal, if at all.

One particular difficulty with trying to explain timeframes around healing was that in many cases nurses suggested healing of a VLU does not take a straightforward trajectory over time, and a wound may appear to get worse. Interviewees described patients becoming *'really disheartened'* (005co). There was consensus that there was usually a discernible cause for an ulcer deteriorating, thus the important thing was to identify the cause and address it: *'You can try and look at a reason why it has happened, like infection. And then treat that cause…….and tell them* [patient] *that you've addressed that so there is some hope for them that will work.'* (004cl).

Knowledge and skills

Gaps in knowledge and skills

There is some suggestion that not all community nurses have the necessary knowledge to recognise the need for a comprehensive leg and wound assessment, or the necessary skills to carry out this initial assessment (including ABPI). Interviewees also recognised a lack of skills among some community nurses in relation to the application of compression bandages: *'The next district nurse that's gone in is not competent in bandaging, so they've not been able to receive the treatment.'* (003cl); and the monitoring of compression therapy:

> '[Patient] *who I sent back to the district nurses to look after…..nobody had bothered to put it* [compression bandages] *back on because she didn't really want it on. So nobody had gone through all the things again, why you need to have it, why it's going to help you….nobody had bothered putting it back on because she'd said "Oh I don't really want it".'* (010cl).

Training

Interviewees identified issues with the provision of training to address the gaps in knowledge and skills as being a barrier to the provision of compression therapy. Although training provision was generally considered to be adequate for those working in more specialised

For peer review only - http://bmjopen.bmj.com/site/about/guidelines.xhtml

roles, for community nurses this was not always the case. A minority of interviewees thought that the availability of training was not good, and a majority mentioned access being more of an issue – it could be challenging to take time out to attend training because of workloads: *'The first thing that gets cancelled is training, unfortunately, 'cos obviously the needs of the patient come before training.'* (005co). It was also noted that staff turnover in the community was high, so there was always a need for training. Some positive examples of training were given; a community nurse talked about training offered by her Tissue Viability Team which she described as *'really good….we do it yearly, so we do have leg ulcer training yearly and compression training'* (005co). Various facilitators of training were suggested, for example it was thought that provision of training 'on site' may get around issues of time to attend. Another interesting issue was the kind of 'informal' training that could be gained by nurses shadowing others in leg ulcer clinics, where these were available: *'You could go and spend an afternoon there and it's just that…..The experts are there, great.'* (014co).

Beliefs about consequences

Encouraging compression use

All interviewees thought that frequent reiteration of explanations about compression being the best therapy was important in encouraging patients to continue using it. They talked about patients being able to 'self-manage' their care and compression therapy as a goal to be aimed for, which they perceived could contribute to the wider use of compression. However this *'can be difficult sometimes, but we're trying to encourage that self-caring as well and encourage them to take control over their care'* (002co). One nurse suggested that barriers to self-care involved not only patients but the thinking and attitudes of nurses themselves:

> *'Self-management's quite a new…when I say new, I mean sort of, in the last few years. But it was always, sort of, the nurse came and did your dressing, and the nurse looked after it. And, I think, even for staff who've been around a long time it's hard getting that sort of, out of your head. So, it's about, sort of, changing your whole attitude and going in from the start and with the idea [that the patient will self-manage].'* (006co).

Response to non-adherence

Many interviewees spoke about how their response to a patient who was not adhering to compression therapy was important in terms of whether that person might carry on, or would try compression again in the future: *'We have to ask why and is there anything else we can do to change that or make it better. Try and find the root of the cause of why they're saying no and then take it from there.'* (002co). It was also universally agreed that there will always be patients who will not tolerate or adhere to compression therapy, and that this was another

For peer review only - http://bmjopen.bmj.com/site/about/guidelines.xhtml

point at which there was a need to be sensitive in responding in order to support the patient and keep the door open for them to try compression again in the future:

> *'I think if they are very sure they don't want to, and we do see…we do see a few like that, you just, I mean, you just kind of say that you understand their choice and, of course, it is their choice what they have on and we understand the reasons why. But that they have to also understand that, you know, healing will be delayed, or indeed will be very difficult to achieve without compression, in a nice way.'* (004cl).

Behavioural regulation

Variation in available bandaging systems

Bandaging systems available for use varied geographically, as recommended by local Tissue Viability or Leg Ulcer Teams. When asked whether she was constrained in what compression therapy she could use for a patient based on where a patient lived and the bandage system used there one clinic-based nurse replied: *'I tend to because you just don't want to make life any more difficult'* (003cl). Community nurses sometimes changed the compression type decided upon by specialist services because they are not able to provide it in their geographical area: *'We change them to* [name of compression brand] *because that's what we can…..that's what our ordering system allows us to order'* (012co).

Intentions

We did not map any barriers to this domain. Instead, the majority of interviewees talked positively about local policies and leg ulcer pathways which they worked within, informed by NICE/SIGN. These were described as useful by interviewees and they talked about referring to them if they were stuck about where to go next with a patient. They were thought to be particularly useful for less experienced nurses. Interviewees articulated conviction that compression was the treatment of choice for someone with a VLU (despite the possibility that some nurses may not have the skills to put it into practice):

> *'I think we are very, sort of, strong in the terms that we are very sure…I feel like we, sort of, give quite a lot of confidence to that treatment. Like, we're just very sure that that is the treatment….But I think that helps that we are very clear that we know what the answer is. And the answer is compression.'* (004cl).

DISCUSSION

Unwarranted variation in clinical practices across wound care services has been highlighted previously.[11] This study sought to understand how and why sub-optimal care happens. In doing so we have used the TDF[14 15] to explore influences on the provision of compression therapy for people with VLUs in the UK. Our analysis suggests that sub-optimal compression use is not necessarily due to lack of knowledge of appropriate care pathways, as has been

For peer review only - http://bmjopen.bmj.com/site/about/guidelines.xhtml

described elsewhere.[23] Instead healthcare staff described a number of factors which they perceived made the provision of compression therapy more challenging. These can broadly be divided into two groups: organisational barriers (TDF domains environmental context and resources, knowledge, skills, behavioural regulation); and patient engagement barriers (TDF domains goals, beliefs about consequences).

Organisational barriers

Our findings echo Franks et al.'s[24] suggestion that the workload of community nursing services can impact on whether and how compression therapy is delivered. Rising patient caseloads are presenting growing challenges in community services,[25] due to both an ageing population and to significant decreases in the number of community nurses.[26] In this study, heavy workloads were seen as contributing to delays in initial leg and wound assessment and in monitoring progress. Work pressures could result in patients being attended by nurses without the necessary knowledge and skills to provide and monitor compression; a decline in the number of qualified District Nurses (holders of the Specialist Practitioner Qualification in District Nursing) in community nursing services[27] could be a contributory factor. Pressured workloads contributed to a lack of continuity of care too; perceived as problematic by nurses in this study as well as being a concern of patients who were interviewed about their experience of using compression therapy (Perry C, Atkinson R, Griffiths J, *et al*., 2021, unpublished).

Problems related to the knowledge and skills of healthcare practitioners in leg ulcer care and compression therapy[23 24] and the provision of adequate training[28] have been described. Traditionally, leg ulcer care has been provided through community nursing services. Interviewees in this study generally considered they had the knowledge and skills to manage venous leg ulcers themselves, but they identified lack of skills in other community staff, and lack of access to training. The main issue described was the time to attend training, alongside high staff turnover resulting in a constant need, and it was suggested that training provided 'on site' might help. Leg ulcer clinics where community nurses were encouraged to 'shadow' specialist leg ulcer nurses as a form of informal training, which could be fitted around their workload, were valued highly.

In this study, community nursing interviewees perceived the absence of specifically commissioned community-based specialist leg ulcer services, to which they could send patients, as a barrier to providing optimal leg ulcer care. Although referral to a specialist service may be necessary for complex leg ulcers, the majority of ulcers could arguably be managed in general community services.[29] Rather than referring to a specialist service, it

For peer review only - http://bmjopen.bmj.com/site/about/guidelines.xhtml

may be that if issues relating to delivery models, workloads, skills and training in the community were addressed, appropriate care for patients with venous leg ulcers would be enabled. The National Health Service England (NHSE)-funded National Wound Care Strategy Programme is supporting a number of sites in the implementation of its lower limb wound recommendations to explore service delivery models and their potential for success.[8]

Organisational barriers to the provision of compression therapy related to prescribing were described. There were variations between bandaging systems available in different geographical areas which meant that not all compression therapies were available in all areas. Local restrictions on the types of compression systems available have been noted in other studies.[30] This could lead to difficulties for clinic or hospital-based nurses, whose patients came from different geographical areas, as they needed to be aware of different availabilities and prescribe accordingly, leading to a 'postcode lottery' of compression therapies. Including commissioners in discussions about service provision and feeding back about the impact of commissioning decisions may be useful in this situation.

Issues with prescribing were also noted when nurses were not qualified to prescribe compression therapies for their patients, instead having to request a prescription from a patient's General Practitioner. This could lead to delays in patients starting compression therapy or a temporary break in their treatment regimen, and interviews with patients found evidence of patients not being in compression because they were waiting for prescribed hosiery to arrive (Perry C, Atkinson R, Griffiths J, *et al*., 2021, unpublished).

Patient engagement barriers

A good relationship between patient and health professional is an important foundation for the successful use of compression therapy.[31-33] In our study, nurses spoke of the importance of getting patients 'on board' with their compression therapy, and of supporting them in using it. Explanations were seen as key and it was almost universally thought that a patient who understood the aetiology of their VLU and the mechanism of compression therapy was more likely to continue to adhere to treatment. However it was evident in a study of patients (Perry C, Atkinson R, Griffiths J, *et al*., 2021, unpublished) that there was variation in patients' perceived understanding of the cause of their VLU and that many people had difficulty understanding this topic. There was not a clear relationship between knowledge/understanding and compression use: individuals who did and did not have an understanding of VLU aetiology or how compression worked, were adherent. It is possible that for some individuals the important thing was that explanations were offered: they were

For peer review only - http://bmjopen.bmj.com/site/about/guidelines.xhtml

happy to rely on the recommendation of their nurse. This highlights the importance of the patient/health professional relationship.

Keeping patients ‘on board’ with their treatment was made harder because of the difficulties in explaining how long a VLU may take to heal and in supporting patients if their VLU become worse rather than improving. Patient interviews (Perry C, Atkinson R, Griffiths J, *et al*., 2021, unpublished) confirm that patients wanted to know how long healing might take and became very disheartened if their VLU became worse, thus underlining the importance of these issues being handled carefully by nurses. Encouraging some degree of ‘self-management’ or perhaps more accurately ‘self-care’ was thought to encourage continued compression use, although there is limited evidence of the benefits or risks of this in relation to compression therapy.

Intervention for change

More data were coded to organisational barriers in this study (TDF domains environmental context and resources, knowledge, skills, behavioural regulation) than to patient engagement barriers (TDF domains goals, beliefs about consequences). This could suggest that a multicomponent intervention will be required to support further delivery of compression in the treatment of VLUs. We recognise that rising patient caseloads and related workload pressures will present significant challenges to any attempt to enhance existing leg ulcer services. Regardless, the organisational barriers highlighted in this study are similar to those highlighted by previous research,[11] but crucially this also suggests that there are similar opportunities to develop facilitated approaches to enable community nursing teams to make changes to practice and enhance the quality of patient care delivered.[25] This information can therefore be used to inform future improvement efforts locally.

Our analysis also suggests that there may be traction in addressing the barriers highlighted in relation to promoting appropriate compression use in people with VLUs. To this end, we have been working with one of the health care provider organisations involved in this work to produce an information leaflet which can be used by all staff to explain and promote the use of compression therapy. Strategies to encourage ‘self-management’ or ‘self-care’ would also merit further investigation.

Study limitations

Although staff from the three participating community services were included in the sample, the majority came from one service. However, the issues raised by staff from all three services were similar. The research was conducted during the COVID-19 pandemic. All

For peer review only - http://bmjopen.bmj.com/site/about/guidelines.xhtml

participants were therefore interviewed over the telephone. This may mean that our study participants were those who were naturally more comfortable with and experienced in talking over the telephone.

There were some challenges in using the TDF, as reported by other users.[8] Some data could be coded to different domains, decisions about coding in these instances were made through discussion between the research team. More data were coded into the environmental context and resources domain than any other. As anything that a healthcare professional does could be viewed as dependent on context,[14] the coding to this domain was examined carefully to ensure that it was the best fit. In one instance coding to the TDF domains split up data (on prescribing) that could usefully be considered together. However, this could be put back together in considering the implications of the findings.

CONCLUSIONS

We have presented an exploration of the factors that prevent or promote the appropriate use of compression therapy for people with VLUs, from the perspective of nurses involved in their care. Whilst patient engagement barriers may be easier and quicker to address than some of the organisational barriers identified, unless organisational barriers are also attended to it would seem unlikely that all people who would benefit from compression therapy will receive it.

Acknowledgments

The authors would like to express thanks to Cheryl Lenney (Chief Nurse, Manchester University NHS Foundation Trust), and Alex Barker (Head of Nursing, Manchester Local Care Organisation) who were members of the Project Management Group (PMG). Thank you to our local Principal Investigators, Louise O'Connor (Lead Nurse for Tissue Viability and Infection Prevention, Manchester University NHS Foundation Trust, who was also a member of the PMG), Joanne Taylor (Tissue Viability Service Lead, Rochdale Care Organisation) and Martin Sylvester (Research Nurse, Mid Yorkshire Hospitals NHS Trust). We also thank our patient representative on the PMG, who has contributed to the work from the outset, including development of the protocol through to interpretation of the findings. The University of Manchester Wounds Research Patient and Public Involvement (PPI) Forum made useful comments on the interview schedule. We thank Francisco Espinoza for setting up the online participant registration form, and the nurse managers at participating Trusts who facilitated recruitment of their staff members as participants. Finally we would like to thank all of the

For peer review only - http://bmjopen.bmj.com/site/about/guidelines.xhtml

staff who so generously gave their time to participate in this research during a very difficult period for NHS services.

Contributors

NC conceived the idea and design for the overall project. RAA, JCD, JG, JFL, and PMW contributed to further development of the study design. CP collected the data. CP, RAA, JG and PMW conducted the data analysis. CP created the original draft of the manuscript. All authors (RAA, NC, JCD, JG, JFL, JM, CP, PMW) contributed to the interpretation of study findings, critical revision of the manuscript for important intellectual content and approval of the final manuscript.

Competing interests

None declared.

Funding

This work was supported by National Institute for Health Research (NIHR) Research for Patient Benefit (RfPB) scheme, grant number PB-PG-0817-20002. NC, JCD, PMW and CP are partially funded by the National Institute for Health and Care Research Applied Research Collaboration Greater Manchester. The views expressed in this publication are those of the author(s) and not necessarily those of the National Institute for Health and Care Research or the Department of Health and Social Care.

Data availability statement

Requests for access to data should be addressed to the corresponding author. Data underlying the study findings will be available on reasonable request. Due to the nature of the ethical approval given for the study it may not be possible to share compete interview transcripts, but additional anonymised illustrative quotations may be available.

Disclaimer

The views expressed in this article are those of the authors and not necessarily those of the NHS, National Institute for Health and Care Research, or the Department of Health and Social care.

Ethics approval

Ethical approval was received in 2019 from the London Bridge NHS Research Ethics Committee (Ref 19/LO/0826).

For peer review only

Patient consent for publication

Not required.

REFERENCES

1. National Institute for Health and Care Excellence. *Varicose veins: diagnosis and management. Clinical guideline.* London: National Institute for Health and Care excellence, 2013.

2. Herber OR, Schnepp W, Rieger MA. A systematic review on the impact of leg ulceration on patients' quality of life. *Health Qual Life Outcomes* 2007;5:44.

3. Green J, Jester R. Health related quality of life and chronic venous leg ulceration: part 1. *Br J Community Nurs* 2009;14:S12-S17. doi: 10.12968/bjcn.2009.14.Sup6.45538

4. Green J, Jester R. Health related quality of life and chronic venous leg ulceration: part 2. Br J Community Nurs 2010;15:S12-S17. doi: 10.12968/bjcn.2010.15.Sup1.46906

5. Cullum N, Buckley H, Dumville J, *et al.* Wounds research for patient benefit: a 5-year programme of research. *Programme Grants for Applied Research 2016;*4:1-304.

6. O'Meara S, Cullum N, Nelson EA, *et al.* Compression for venous leg ulcers. *Cochrane Database Syst Rev* 2012;11:CD000265.

7. Scottish Intercollegiate Guidelines Network. *Management of chronic venous leg ulcers*: *A national clinical guideline (120):* Scottish Intercollegiate Guidelines Network, 2010.

8. National Wound Care Strategy Programme. *Improving Wound Care.* Accessed 12/01/22 https://www.nationalwoundcarestrategy.net/improving-wound-care/

9. Ashby RL, Gabe R, Ali S, *et al.* VenUS IV (Venous leg Ulcer Study IV) - compression hosiery compared with compression bandaging in the treatment of venous leg ulcers:

For peer review only - http://bmjopen.bmj.com/site/about/guidelines.xhtml

a randomised controlled trial, mixed-treatment comparison and decision-analytic model. *Health Technol Assess* 2014;18:1-294.

10. Nelson EA, Bell-Syer SEM. Compression for preventing recurrence of venous ulcers. *Cochrane Database Syst Rev* 2012;8:CD002303.

11. Gray TA, Rhodes S, Atkinson RA, *et al.* (2018). Opportunities for better value wound care: a multiservice, cross-sectional survey of complex wounds and their care in a UK community population. *BMJ Open* 2018;8:e019440. doi:10.1136/bmjopen-2018-024859

12. Phillips P, Lumley E, Duncan R, *et al.* A systematic review of qualitative research into people's experiences of living with leg ulcers. *J Adv Nurs* 2018;74:550–563. doi: 10.1111/jan.13465

13. Weller CD, Buchbinder R, Johnston RV. Interventions for helping people adhere to compression treatments for venous leg ulceration. *Cochrane Database Syst Rev* 2016;3:CD008378.

14. Atkins L, Francis J, Islam R, *et al.* A guide to using the Theoretical Domains Framework of behaviour change to investigate implementation problems. *Implement Sci* 2017;12:77. doi: 10.1186/s13012-017-0605-9

15. Cane J, O'Connor D, Michie S. Validation of the theoretical domains framework for use in behaviour change and implementation research. *Implement Sci* 2012;7:37.

16. Francis JJ, Johnston M, Robertson C, *et al.* What is an adequate sample size? Operationalising data saturation for theory-based interview studies. *Psychol Health* 2010;25:1229-45.

17. Denscombe M. The good research guide for small scale social research projects. Philadelphia: Open University Press 2003:(2nd ed).

18. Bryman A. Social research methods. Oxford United Kingdom: Oxford University Press 2008: (3rd ed).

For peer review only - http://bmjopen.bmj.com/site/about/guidelines.xhtml

19. Britten N. Qualitative research. Qualitative interviews in medical research. *BMJ* 1995;311:251-253.

20. Gibbs G. Analyzing qualitative data. London United Kingdom: Sage 2007.

21. Ritchie J, Spencer L, O'Connor W. (2003). Carrying out qualitative analysis. In: Ritchie J, Lewis J, eds. Qualitative research in practice: A guide for social science students and researchers. CA: Sage 2003:220-262.

22. Tobin GA, Begley CM. Methodological rigour within a qualitative framework. *J Adv Nurs* 2004;48:388–396.

23. Weller CD, Richards C, Turnour AM, *et al.* Barriers and enablers to the use of venous leg ulcer clinical practice guidelines in Australian primary care: A qualitative study using the theoretical domains framework. *Int J Nurs Stud* 2019;103.

24. Franks PJ, Barker J, Collier M, *et al.* Management of patients with venous leg ulcers. Challenges and current best practice. *J Wound Care* 2016;25:s1-s67.

25. Swift A, Punshon G. *District Nursing Today. The view of District Nurse Team Leaders in the UK.* London: The Queen's Nursing Institute, International Community Nursing Observatory, 2019.

26. Nuffield Trust. *The NHS workforce in numbers. Facts on staffing and staff shortages in England.* Accessed 09/11/21www.nuffieldtrust.org.uk/resource/the-nhs-workforce-in-numbers

27. Queen's Nursing Institute. *Report on District Nurse Education in the United Kingdom 2017 – 2018.* London: The Queen's Nursing Institute, 2019.

28. Collaboration for Leadership in Applied Health Research and Care Greater Manchester. *ILUMIN: The Leg Ulcer Quality Improvement Programme Summary Report.* Collaboration for Leadership in Applied Health Research and Care Greater Manchester, 2019.

29. National Institute for Health and Care Excellence. *Overview of management of a person with a venous leg ulcer.* Accessed 30/11/21https://cks.nice.org.uk/topics/leg-ulcer-venous/management/venous-leg-ulcers/#when-to-refer

For peer review only - http://bmjopen.bmj.com/site/about/guidelines.xhtml

30. Oates O, Adderley U. Survey of registered nurses' selection of compression systems for the treatment of venous leg ulcers in the UK. *J Tissue Viability* 2019;28:115–119. doi.org/10.1016/j.jtv.2019.02.004

31. Annells M, O'Neill J, Flowers C. Compression bandaging for venous leg ulcers: the essentialness of a willing patient. *J Clin Nurs* 2008;17:350-359. doi: 10.1111/j.1365-2702.2007.01996.x

32. Douglas V. Living with a chronic leg ulcer: an insight into patients' experiences. *J Wound Care* 2001;10:355-360.

33. Van Hecke A, Verhaeghe S, Grypdonck M, *et al.* Processes underlying adherence to leg ulcer treatment: A qualitative field study. *Int J Nurs Stud* 2011;48:145-155.

For peer review only

For peer review only - http://bmjopen.bmj.com/site/about/guidelines.xhtml

Barriers and facilitators to compression therapy for people with venous leg ulcers – staff interview schedule

As part of this project we're really interested in understanding the barriers and facilitators to the delivery of compression therapy for people with venous leg ulcers, including factors that may operate at an individual, team and organisation level. We are also interested in your perspectives on how the behaviours of patients themselves might impact positively or negatively on compression use.

Question	Prompts	Rationale/Theoretical Domains Framework (TDF) domains
**Can you tell me a little bit about your current role and your main responsibilities?**	• *How long have you been in this role?*<br>• *What did you do before?*<br>• *How is your team/clinic set up and organised?*	Easy first question, scene setting, supplements demographic information supplied, may reveal something important in relation to role which can be probed later (e.g. 'go to' leg ulcer person).
**From your perspective, is there a 'typical' leg ulcer patient? Could you describe them to me?**	• *Age/sex?*<br>• *Co-morbidities?*<br>• *Lifestyle factors?*	Scene setting.
**Can you tell me what 'gold standard' care and treatment for a patient with a venous leg ulcer looks like?**	• *Vascular assessment.*<br>• *Cleansing and debridement.*<br>• *Dressings and topical treatments.*<br>• *Compression therapy.*<br>• *Systemic therapy.*<br>• *Provision of care.*	Establishing something about interviewees' knowledge/experience – the background against which they practice.<br><br>TDF: knowledge; professional role and identity.
**What guides your choices about venous leg ulcer care?**	• *Knowledge and skills – training, peers, experience, preferences?*<br>• *Local policies?*	TDF: knowledge; skills; professional role and identity; beliefs about capabilities; optimism; beliefs about consequences;

For peer review only - http://bmjopen.bmj.com/site/about/guidelines.xhtml

Question	Prompts	Rationale/Theoretical Domains Framework (TDF) domains
	• *Research evidence - Scottish Intercollegiate Guidelines Network (SIGN) guidelines, Cochrane reviews, reading journals, online searches, specialist peers?*<br>• *Patient and carers' influence – lifestyle, preference, adherence.*	reinforcement; intentions; memory, attention and decision processes; environmental context and resources; social influences; emotion; behavioural regulation.
**What is your experience of offering and delivering compression therapy to patients with venous leg ulcers?**	• *How important do you think compression is?*<br>• *What guides your decision to offer compression? What are your thought processes?*<br>• *How do you explain it to patients and what information do you routinely give them (any patient information/leaflets given)?*<br>• *Do you think patients understand the importance of it?*<br>• *Is there a particular type of compression you like?*<br>• *Do you start on full compression and reduce if patient cannot tolerate/start low and work up to full? Why?*<br>• *How easy or difficult do you find it to deliver compression therapy?*<br>• *What do you think are the necessary skills for effective compression delivery?*<br>• *Have you received relevant training to deliver compression therapy?*<br>• *Are there times when you would choose not to offer or deliver compression (even if it was clinically indicated)?*	TDF: knowledge; skills; professional role and identity; beliefs about capabilities; optimism; beliefs about consequences; reinforcement; intentions; goals; memory, attention and decision processes; environmental context and resources; social influences; emotion; behavioural regulation.
**I'd like to ask you specifically about what you see as the barriers to offering and delivering compression therapy, and what the facilitators are.**<br><br>**Are barriers and facilitators to offering compression therapy different to those**	***Individual level***<br>• *Do you personally find it easy/difficult to offer and deliver compression therapy?*<br>• *How important do you think the ABPI measurement is?*<br>• *What do you do if you are looking after a patient who has not had an ABPI measurement taken?*<br>• *How confident are you in using a Doppler?*	TDF: knowledge; skills; beliefs about capabilities; emotion.

For peer review only

Question	Prompts	Rationale/Theoretical Domains Framework (TDF) domains
**of delivering compression therapy (may want to address separately if so)?**<br><br>**It may be easier to look at barriers and facilitators in turn. Barriers? Facilitators?**	• *How confident are you in offering and delivering compression therapy?*<br>• *Is there anything that would make you more confident?*<br><br>***Team level***<br>• *Would any other team members influence whether or not you offer or deliver compression therapy (who and how)?*<br>***Organisational level***<br>• *Are there aspects of the community/clinic environment which influence whether or not you are able to offer and deliver compression?*<br>• *Are there any time constraints that influence whether or not you are able to offer and deliver compression?*<br>• *Are there any competing tasks that influence whether or not you are able to offer and deliver compression?*<br>• *Are there any specific issues (e.g. staffing) that influence whether or not you are able to offer and deliver compression?*<br>*If yes to any of the above – how big an issue is this, how does this issue affect your decision making, do you use simpler techniques/interventions so that less experienced staff can then continue using them?*<br>• *What systems are in place to assist with compression use (how easy or difficult is it to deliver compression)?*<br>• *What availability is there of Dopplers; staff training; patient information leaflets?*	TDF: professional role and identity; reinforcement; social influences.<br>TDF: professional role and identity; reinforcement; intentions; environmental context and resources; social influences; behavioural regulation.
**How might the views of your patients affect how you offer and deliver compression?**	• *Does compression therapy influence your relationships with patients? (does it affect communication?)*<br>• *How do you respond when a patient refuses to wear compression/is non-adherent?*<br>• *Do your colleagues generally agree with you about this approach to patient non-adherence?*	TDF: beliefs about consequences; goals; environmental context and resources; social influences; emotion.

For peer review only - http://bmjopen.bmj.com/site/about/guidelines.xhtml

Question	Prompts	Rationale/Theoretical Domains Framework (TDF) domains
	• *Do you find that you change from full compression to reduced compression?*<br>• *What are your views about reduced compression?*<br>• *Do you find patients adhere to reduced compression?*<br>• *Does not being able to deliver compression cause you to be worried or concerned?*	
**Have you any views on why patients do or do not adhere to their compression therapy?**	• *Patient discomfort?*<br>• *Patient lifestyle?*<br>• *Patient beliefs about illness and treatment?*<br>• *Influence of partner/family/friends?*<br>• *Do your colleagues generally agree with you about patient non-adherence?*	TDF: beliefs about consequences; goals; environmental context and resources; social influences; emotion.
**What do you perceive to be the consequences of offering and delivering compression therapy?**	• *Does it influence the outcomes?*<br>• *Does it have an impact on your clinical workload and patient routines?*<br>• *What happens if you are not able to deliver compression as you would like to? (to patients, to colleagues, to yourself? Short and long-term consequences?)*	TDF: beliefs about consequences; optimism; intentions; goals; social influences; emotion.
**What would help you to overcome any difficulties/challenges that you perceive in offering and delivering compression therapy?**	• Skills training in using a Doppler machine?<br>• Other skills training?<br>• The number of Dopplers available?<br>• Other equipment?<br>• *What would you personally have to do to increase the number of patients wearing compression? (in the ideal world).*<br>• *If you wanted to implement changes in your own practice to increase the number of patients wearing compression, what would be some of the ways to do this?*	TDF: knowledge; skills; beliefs about capabilities; optimism; reinforcement; memory, attention and decision processes; environmental context and resources; behavioural regulation.

For peer review only - http://bmjopen.bmj.com/site/about/guidelines.xhtml

Question	Prompts	Rationale/Theoretical Domains Framework (TDF) domains
**Is there anything else about your experience of offering and delivering compression therapy that I haven't asked you about that you think it is important to say?**		Tapping into knowledge/experience of individual.
**Is there anybody else that you can think of (individual/groups) who you think it might be useful for me to talk to as part of this study?**		Snowballing/tapping into knowledge/experience of individual.

For peer review only

For peer review only - http://bmjopen.bmj.com/site/about/guidelines.xhtml

COREQ (Consolidated criteria for Reporting Qualitative research) Checklist

Topic	Item No.	Guide Questions/Description	Reported on Page No.
**Domain 1: Research team and reflexivity**			
Personal characteristics			
Interviewer/facilitator	1	Which author/s conducted the interview or focus group?	7 (resubmitted manuscript)
Credentials	2	What were the researcher's credentials? E.g. PhD, MD	Supplementary material below
Occupation	3	What was their occupation at the time of the study?	Supplementary material below
Gender	4	Was the researcher male or female?	Supplementary material below
Experience and training	5	What experience or training did the researcher have?	Supplementary material below
Relationship with participants			
Relationship established	6	Was a relationship established prior to study commencement?	Supplementary material below
Participant knowledge of interviewer	7	What did the participants know about the researcher? E.g. personal goals, reasons for doing the research.	Supplementary material below
Interviewer characteristics	8	What characteristics were reported about the interviewer/facilitator? E.g. bias, assumptions, reasons and interests in the research topic.	Supplementary material below
**Domain 2: Study design**			
Theoretical framework			
Methodological orientation and theory	9	What methodological orientation was stated to underpin the study? E.g. grounded theory, discourse analysis, ethnography, phenomenology, content analysis.	7 (resubmitted manuscript)
Participant selection			
Sampling	10	How were participants selected? E.g. purposive, convenience, consecutive, snowball.	7 (resubmitted manuscript)
Method of approach	11	How were participants approached? E.g. face-to-face, telephone, mail, email.	7 (resubmitted manuscript)
Sample size	12	How many participants were in the study?	8 (resubmitted manuscript)
Non-participation	13	How many people refused to participate or dropped out? Reasons?	7 (resubmitted manuscript) and supplementary material below
Setting			
Setting of data collection	14	Where was the data collected? E.g. home, clinic, workplace.	7 (resubmitted manuscript)
Presence of non-participants	15	Was anyone else present besides the participants and researchers?	N/A

For peer review only - http://bmjopen.bmj.com/site/about/guidelines.xhtml

Topic	Item No.	Guide Questions/Description	Reported on Page No.
Description of sample	16	What are the important characteristics of the sample? E.g. demographic data, date.	7 and 8-10 (resubmitted document)
Data collection			
Interview guide	17	Were questions, prompts, guides provided by the authors? Was it pilot tested?	7 (resubmitted document) and Supplementary File 1
Repeat interviews	18	Were repeat interviews carried out? If yes, how many?	N/A
Audio/visual recording	19	Did the research use audio or visual recording to collect the data?	7 (resubmitted document)
Field notes	20	Were field notes made during and/or after the interview or focus group?	Supplementary material below
Duration	21	What was the duration of the interviews or focus group?	8 (resubmitted document)
Data saturation	22	Was data saturation discussed?	7 (resubmitted document)
Transcripts returned	23	Were transcripts returned to participants for comment and/or correction?	Supplementary material below
**Domain 3: analysis and findings**			
Data analysis			
Number of data coders	24	How many data coders coded the data?	7-8 (resubmitted document)
Description of the coding tree	25	Did the authors provide a description of the coding tree?	11 (resubmitted document)
Derivation of themes	26	Were themes identified in advance or derived from the data?	7 (resubmitted manuscript)
Software	27	What software, if applicable, was used to manage the data?	7 (resubmitted manuscript)
Participant checking	28	Did participants provide feedback on the findings?	8 (resubmitted document)
Reporting			
Quotations presented	29	Were participant quotations presented to illustrate the themes/findings? Was each quotation identified? E.g. participant number.	11 (resubmitted manuscript) and throughout results
Data and findings consistent	30	Was there consistency between the data presented and the findings?	Throughout results
Clarity of major themes	31	Were major themes clearly presented in the findings?	Throughout results
Clarity of minor themes	32	Is there a description of diverse cases or discussion of minor themes?	Throughout results

For peer review only - http://bmjopen.bmj.com/site/about/guidelines.xhtml

Developed from: Tong A, Sainsbury P, Craig J. Consolidated criteria for reporting qualitative research (COREQ): a 32 item checklist for interviews and focus groups. *International Journal for Quality in Health Care.* 2007. Volume 19, Number 6: pp.349-357.

Supplementary material
Below is presented additional detail about issues that it was not possible to present in the main paper, arranged under the COREQ headings.

Research team and reflexivity
Please see page 7 (resubmitted paper), with supplementary material below.

Personal characteristics
Interviews were conducted by one researcher (CP). She was female, held a PhD, and was an experienced interviewer who had received training in qualitative research methods over a number of years, including during the conduct of her PhD. At the time of the study the researcher was employed as a University Research Fellow.

Relationship with participants
There was no relationship between research participants and the researcher prior to the commencement of the study. Potential participants had been provided with details of the study by their manager and if they were interested in learning more had completed an on-line form. They were then contacted via email by the researcher. The researcher's role as a University researcher was explained to individual potential interviewees. It was ensured that researcher contact details were clearly available in case potential participants had further questions.

Study design
Please see main pages 7-8 (resubmitted paper), with supplementary material below.

Method of approach
We liaised with the nurse managers to ensure that they used a standardised text to approach their staff, this text was approved by the NHS Ethics Committee who gave ethical approval for the research. The nurse managers sent out the invite only once and did not follow it up, and would not know who did or did not respond to the invitation.

Participant selection
Eighteen potential participants registered their interest in participating in the study; three of these people did not respond to interview invitations and so were not interviewed.

Data collection
We developed a semi-structured interview schedule (Supplementary File 1) with reference to the research aim, the literature, and the Theoretical Domains Framework. This schedule was 'sense checked' by practitioner members of the study team. The interview schedule was kept under review as the interviews progressed to ensure that we were generating relevant data. Interview participants were offered sight of transcripts, although nobody availed themselves of this. Field notes were used to provide context.

Analysis and findings
Please see pages 7-11 (resubmitted paper) and throughout results.

For peer review only - http://bmjopen.bmj.com/site/about/guidelines.xhtml
